# Polymorphism and Phase Stability of Hydrated Magnesium Carbonate Nesquehonite MgCO_3_·3H_2_O: Negative Axial Compressibility and Thermal Expansion in a Cementitious Material

**DOI:** 10.1021/acs.cgd.3c01171

**Published:** 2024-01-24

**Authors:** David Santamaría-Pérez, Raquel Chuliá-Jordán, Javier Gonzalez-Platas, Alberto Otero-de-la-Roza, Javier Ruiz-Fuertes, Julio Pellicer-Porres, Robert Oliva, Catalin Popescu

**Affiliations:** † Departamento de Física Aplicada-ICMUV, MALTA Consolider Team, Universitat de València, Valencia 46100, Spain; ‡ Departamento de Didáctica de las Ciencias Experimentales y Sociales, Universitat de Valencia, Valencia 46022, Spain; § Departamento Física, Instituto Universitario de Estudios Avanzados en Física Atómica, Molecular y Fotónica (IUDEA), MALTA Consolider Team, Universidad de La Laguna, Tenerife 38204, Spain; ∥ Departamento de Química Física y Analítica, Facultad de Química, MALTA Consolider Team, Universidad de Oviedo, Oviedo 33006, Spain; ⊥ DCITIMAC, MALTA Consolider Team, Universidad de Cantabria, Santander 39005, Spain; # GEO3BCN−Geosciences Barcelona, CSIC, Barcelona 08028, Spain; ∇ CELLS-ALBA Synchrotron Light Facility, Cerdanyola del Vallés, Barcelona 08290, Spain

## Abstract

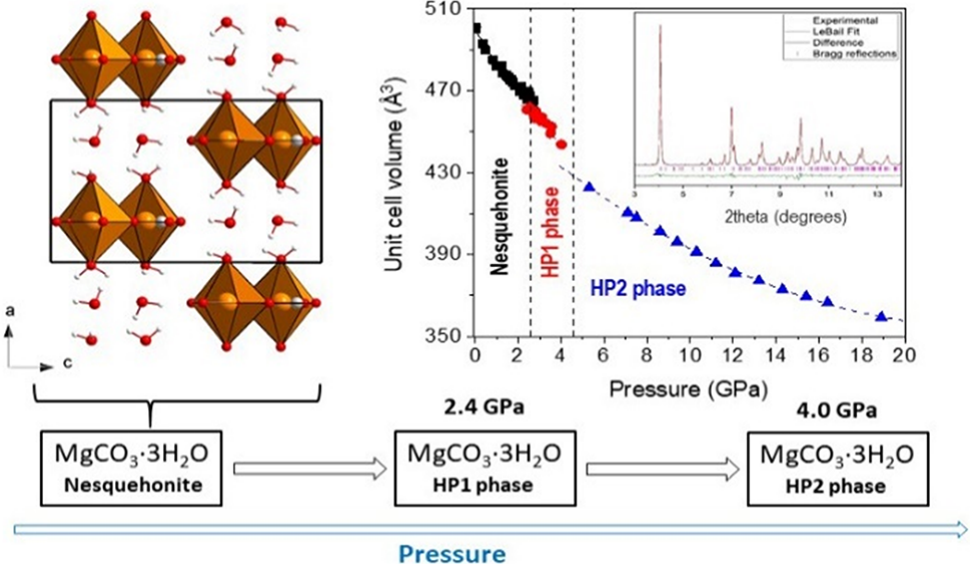

The *P*–*T* phase diagram of the hydrated magnesium carbonate nesquehonite (MgCO_3_·3H_2_O) has not been reported in the literature. In this paper, we present a joint experimental and computational study of the phase stability and structural behavior of this cementitious material at high-pressure and high-temperature conditions using *in situ* single-crystal and synchrotron powder X-ray diffraction measurements in resistive-heated diamond anvil cells plus density functional theory calculations. Our results show that nesquehonite undergoes two pressure-induced phase transitions at 2.4 (HP1) and 4.0 GPa (HP2) at ambient temperature. We have found negative axial compressibility and thermal expansivity values, likely related to the directionality of the hydrogen bonds. The equations of state of the different phases have been determined. All the room-temperature compression effects were reversible. Heating experiments at 0.7 GPa show a first temperature-induced decomposition at 115 °C, probably into magnesite and a MgCO_3_·4H_2_O phase.

## Abstract 2

The high-pressure high-temperature phase stability and structural behavior of hydrated magnesium carbonate nesquehonite (MgCO_3_·3H_2_O) have been studied using *in situ* single-crystal and synchrotron powder X-ray diffraction measurements in resistive-heated diamond anvil cells and DFT calculations. Nesquehonite has negative axial compressibility and thermal expansivity, and it undergoes two pressure-induced phase transitions at 2.4 and 4.0 GPa at ambient temperature. A novel dehydration product at 0.7 GPa and 115 °C is reported.

## Introduction

Mineral carbonation is one of the most promising technologies for long-term carbon dioxide sequestration.^1−4^ Many of the scientific approaches to CO_2_ sequestration rely on the reactions between CO_2_ and certain divalent cations (M^2+^) with the expected formation of M-carbonate minerals as final products. Realistically available choices are largely confined to alkaline earth Ca and Mg metals due to their natural abundance and the stability of the corresponding carbonates. Magnesium silicates, such as olivine and serpentine, are more abundant than the Ca-bearing silicates, like wollastonite, so Mg sources are readily available. Therefore, magnesium carbonates offer attractive possibilities for permanent storage of CO_2_.^5,6^

Magnesium carbonates exist in a wide variety of naturally occurring minerals within the MgO–CO_2_–H_2_O system: the anhydrous magnesite MgCO_3_,^7^ the hydrated barringtonite MgCO_3_·2H_2_O,^8^ nesquehonite MgCO_3_·3H_2_O,^9^ lansfordite MgCO_3_·5H_2_O,^10^ basic hydromagnesite Mg_5_(CO_3_)_4_(OH)_2_·4H_2_O,^11^ protohydromagnesite Mg_5_(CO_3_)_4_(OH)_2_·11H_2_O,^12^ an unnamed mineral Mg_5_(CO_3_)_4_(OH)_2_·8H_2_O,^13^ dypingite and giorgiosite Mg_5_(CO_3_)_4_(OH)_2_·5H_2_O,^14,15^ and artinite Mg_2_(CO_3_)(OH)_2_·3H_2_O.^16^ On top of that, a new hydrate of magnesium carbonate, MgCO_3_·6H_2_O, has been recently synthesized in the laboratory.^17^ Aqueous-phase carbonation reactions yield hydrated magnesium carbonates, and nesquehonite is the most commonly observed hydrated phase near ambient conditions.^18^ For instance, this solid can be obtained by reacting CO_2_ from industrial gas streams and magnesium from desalination brines in alkaline environments.^19^ Free energy calculations of magnesium carbonates and hydrates at ambient CO_2_ and H_2_O partial pressures indicate that nesquehonite is the most stable phase at low temperatures (*T* < 269 K), with magnesite becoming more stable above that temperature.^20^ The formation of the nesquehonite mineral was explained in terms of the high hydration energy of the Mg^2+^ ions in solution that would kinetically inhibit the formation of the anhydrous magnesite.^20^

Nesquehonite has been typically reported as a magnesium carbonate with 3 water molecules included in the structure and a formula unit that can be written as MgCO_3_·3H_2_O.^9,21,22^ However, other studies have suggested that the chemical formula should be redefined as Mg(HCO_3_)(OH)·2H_2_O, with bicarbonate and hydroxyl groups existing in the structure.^23,24^ This controversy was recently solved by accounting for differences in formation conditions, the Mg(HCO_3_)(OH)·2H_2_O compound crystallizing in a solution with pH < 8 while MgCO_3_·3H_2_O nesquehonite occurring at pH between 8.5 and 12.5.^22,25^

**Figure 1:**
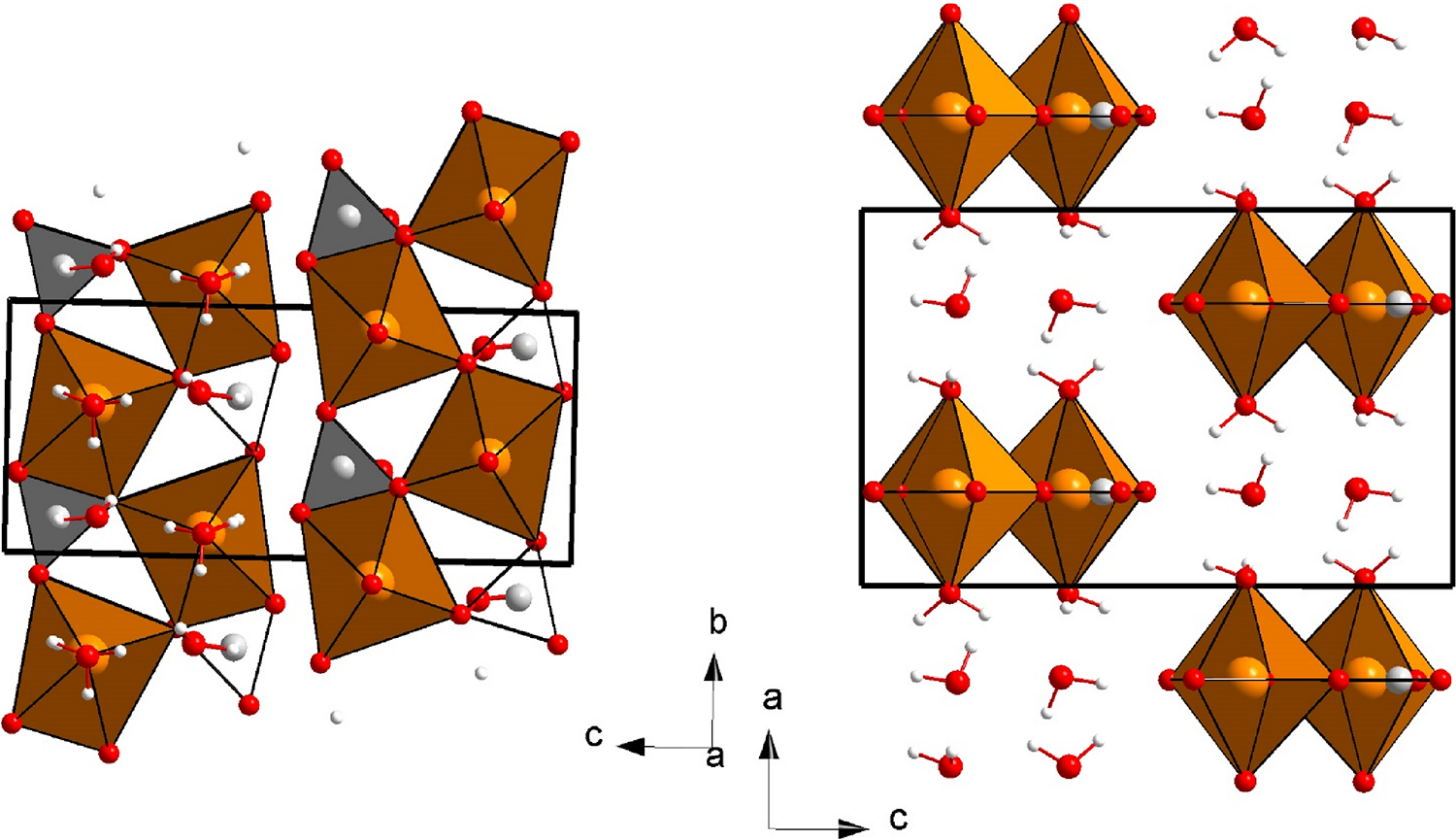
Projections of the structure of MgCO_3_·3H_2_O nesquehonite at room conditions perpendicular to the *a* axis (left) and the *b* axis (right). Orange, red, gray, and white spheres correspond to the Mg, O, C, and H atoms, respectively. Cation-centered oxygen polyhedra are also depicted.

Despite its occurrence in nature and its possible implications on CO_2_ sequestration as a magnesium carbonate mineral, nesquehonite has not attracted much attention.^26,27^ The crystal structure of MgCO_3_·3H_2_O nesquehonite was first reported by Stephan and MacGillavry in 1972.^9^ It was described with a *P*2_1_/*n* monoclinic unit cell and consists of strongly distorted [MgO_6_] octahedra that share an equatorial edge with a carbonate group and the other two equatorial corners with a carbonate group each (Figure 1). The apical oxygen atoms surrounding the Mg atoms come from two water molecules.^9,21,22^ As a result, [MgO_6_] and [CO_3_] units are linked forming complex chains parallel to the *b* direction. The third noncoordinating water molecule is located between chains. Several groups have reported thermal analyses of nesquehonite at room pressure and found three temperature decomposition stages between 52 and 250 °C, but the high-temperature phases with a lower H_2_O content could not be fully identified.^26−28^ Interestingly, thermally activated nesquehonite can easily be regenerated in the presence of water,^27^ which potentially offers a thermally regenerable self-cementation system. However, despite the attractive physical properties of this compound as a CO_2_ sequestration product via mineralization with potential useful applications as a construction material, no studies on the phase stability of nesquehonite under compression, either at room or high temperature, are available in the literature.

In order to give further insights into the polymorphism and decomposition of MgCO_3_·3H_2_O nesquehonite at high pressure (HP) and high temperature (HT), we have undertaken a joint experimental and computational investigation of its phase stability and structural properties. We have characterized it *in situ* using single-crystal and synchrotron powder X-ray diffraction using resistive-heated diamond anvil cells (DACs) and found two phase transitions at 2.4(1) and 4.0(3) GPa under compression at room temperature. We also confirmed the existence of a novel hydrated carbonate under compression and heating. The compressibility, thermal expansion coefficient, and decomposition temperatures upon compression were determined. Density functional theory (DFT) calculations were used to complement the experimental results.

## Experimental Details

A synthetic white MgCO_3_·3H_2_O nesquehonite sample was provided by the Yale Peabody Museum (specimen YPM MIN 031567). This sample was prepared by Genth and Penfield more than a century ago.^29^ It consisted of small single crystals and powder characterized by grains ∼1 μm in size. Chemical analyses were done on a Philips XL30 scanning electron microscope using energy-dispersive X-ray spectroscopy. Only traces (<0.5 at. %) of Mn and Fe were found in addition to the Mg, C, and O atoms present in the ideal MgCO_3_·3H_2_O nesquehonite composition. XRD and Raman measurements at ambient conditions confirmed that the sample has the previously reported structure.^9^

Two HP angle-dispersive single-crystal XRD experiments were performed at room temperature using a Rigaku SuperNOVA diffractometer equipped with an EOS CCD detector and a Mo radiation microsource (λ = 0.71073 Å). All measurements were processed with CrysAlis software version 1.171.42.72a.^30^ Numerical absorption correction based on Gaussian integration over a multifaceted crystal model was applied using the ABSORB-7 program.^31^ For these HP measurements, we have used a Mini-Bragg DAC from Almax-EasyLab, with an opening angle of 85° and anvil culets of 500 μm diameter, fitted with a preindented stainless-steel gasket containing a hole 200 μm in diameter and 70 μm in depth. In the first HP experiment, we used a 4:1 methanol–ethanol mixture as a pressure transmitting medium (PTM), while in the second, the PTM was Paratone oil. In each case, the sample was placed on one of the diamonds anvils together with a small ruby sphere as a pressure sensor.^32^ The structure was refined, for each pressure, using previous results as the starting point, on F^2^ by full-matrix least-squares refinement using the SHELXL program.^33^ Due to limitations of the opening angle of our DAC, it is only possible to collect about 35% of the reflections present in a full data set for the monoclinic space group at ambient conditions.

Two HP angle-dispersive powder XRD experiments at room temperature were conducted at the MSPD beamline of the ALBA-CELLS Synchrotron Light Source^34^ with an incident monochromatic wavelength of 0.4246 Å focused to 20 × 20 μm^2^. These measurements were performed in a membrane-type DAC with 500 μm diamond culets. Nesquehonite powder was loaded in a 160 μm-diameter hole of an Inconel gasket preindented to a thickness of about 50 μm. Two PTMs were used: (i) high-purity Ne gas was loaded in the DAC by means of a Sanchez Technologies gas loading apparatus, providing a fluid environment up to 4.7 GPa at room temperature^35^ and a quasi-hydrostatic medium below 15 GPa,^36^ and (ii) silicone oil, which assures relative small nonhydrostatic stresses in the pressure chamber up to 10.5 GPa.^36^ Pressure was measured based on the ruby fluorescence technique^32^ and the Cu equation of state (EOS).^37^

Finally, an HP-HT powder XRD experiment was conducted at the same synchrotron beamline. In this run, the pressure was increased up to 0.7 GPa, and then, the sample was progressively heated at a 1 °C/min rate. The dehydration and decomposition phenomena were characterized *in situ* using a resistive-heating system for membrane-type DACs. Our DAC had diamond culets of 400 μm, and the pressure chamber had dimensions of 150 μm diameter and 40 μm thickness. The HP-HT system consists of a heating ring that heats all the parts of the DAC to apply a homogeneous temperature at the sample up to 160 °C. The temperatures were measured using a K-type thermocouple attached to one of the diamond anvils, close to the gasket.^38,39^ NaCl powder was included in the sample chamber to act as a pressure marker,^40^ and silicone oil was used as a PTM.

Detector calibration, correction of distortion, and integration to conventional 2θ-intensity powder XRD data were carried out with Dioptas software.^41^ The indexing and refinement of the powder patterns were performed using the Unitcell,^42^ Powdercell,^43^ and Fullprof^44^ program packages.

## Computational Details

Density functional calculations (DFT) of the nesquehonite phase were carried out in the pseudopotentials/plane-wave approach using the projector-augmented wave (PAW) method^45^ as implemented in the Quantum ESPRESSO^46^ package (version 6.5). We used the B86b exchange^47^ and PBE correlation^48^ functionals (B86bPBE) together with the exchange-hole dipole moment (XDM) dispersion model.^49−51^ This methodology has successfully been used to study the phase diagrams of numerous carbonate systems.^52,53^ The PAW data sets used from the pslibrary^54^ had 1 (H), 4 (C), 6 (O), and 10 (Mg) valence electrons. The energy cutoffs and *k*-point grid density were determined by examining the convergence of the total energy (to around 0.1 mRy) and the calculated pressure (0.01 GPa). Energy cutoffs of 100 and 1000 Ry were used for the Kohn–Sham state and electron density plane-wave expansions, respectively. Reciprocal space integrations in the nesquehonite phase were carried out using a uniform 2 × 3 × 2 *k*-point grid.

We found the equilibrium geometry of the nesquehonite phase at 0 and 50 GPa. In all geometry relaxations, tight convergence criteria (10^–4^ Ry/bohr in the forces and 10^–5^ in the energies) were used. The resulting equilibrium volumes were used to build a uniform volume grid with 41 points. At each of these points, constant-volume geometry relaxations were carried out. Once the relaxations were done, we performed density functional perturbation theory (DFPT) calculations^55^ at each equilibrium geometry in the volume grid to obtain the harmonic vibrational frequencies, in order to calculate the vibrational contribution to the free energy. The DFPT calculations used a 2 × 2 × 2 uniform *q*-point grid in reciprocal space. The resulting phonon density of states at each volume, together with the electronic energy, was passed to the gibbs2^56,57^ program that uses the quasi-harmonic approximation to calculate the system’s volume at an arbitrary temperature and pressure, as well as other thermodynamic properties.

## Results

### Crystal Structure Refinement of Nesquehonite at Room Conditions

The crystallographic data of the nesquehonite sample at room conditions have already been published,^9,21,22^ and the structure has been described with the monoclinic system in the *P*2_1_/*n* space group. Our single-crystal measured data have allowed refining the structure at pressures between room pressure and 2.8 GPa using the SHELXL program. Table S1, in the Supporting Information, collects the lattice parameters and atomic coordinates at room conditions from our experiment and DFT calculations, together with those previously obtained by single-crystal XRD^21^ and neutron powder diffraction^22^ for the sake of comparison. As can be seen, there is good agreement. It should be mentioned that, applying the ADDSYM procedure in PLATON software,^58^ it is obtained that the structure can be described in the orthorhombic group *Pnma*. The symmetry of the refined coordinate set is indeed very close to *Pnma*, and the Laue averaging *R*-value to *mmm* (*R*(int)) is also very low. However, the *a*-glide extinction condition is not very satisfactory as can be gleaned from the list of outliers that are listed in the lst file, and therefore, the space group *P*2_1_/*n* provides a better description. As hydrogen atoms are weak X-ray scatterers, all hydrogen atoms have been placed in geometrically suitable positions and refined with an isotropic thermal parameter related to the equivalent isotropic thermal parameter of the parent atom (Uiso(H) = 1.5 Ueq(O)). The geometrical analysis of interactions in the structure was performed with the PLATON program.^58^ The adequacy of the hydrogen bonding network observed in neutron diffraction experiments determines, for instance, the final space group choice. Therefore, we maintained the consideration that this compound is monoclinic at *T* = 293(2) K and 1 atm. The atomic coordinates can also be found in the cif file provided as the Supporting Information.

The crystal structure of nesquehonite is represented in Figure 1 and can be described as formed by double chains of [CO_3_] trigonal and highly distorted [MgO_6_] octahedral groups parallel to the *b* axis. The distortion of the Mg-centered octahedra comes from the fact that they share an edge with a carbonate group, which entails a very short O–O equatorial distance for a [MgO_6_] octahedron (2.17 vs 3.135 Å, the average of the other O–O equatorial distances, and 2.939 Å, the average of all the equatorial-axial O–O distances). The equatorial O atoms of the [MgO_6_] units are part of 3 [CO_3_] carbonate groups, and the axial O atoms belong to water molecules. The rest of the H_2_O molecules lie between these double chains, connecting them by means of hydrogen bonds. In other words, the nesquehonite structure possesses a unique combination of covalent, ionic, and hydrogen bonds.

### Nesquehonite under Room-Temperature Compression

**Figure 2:**
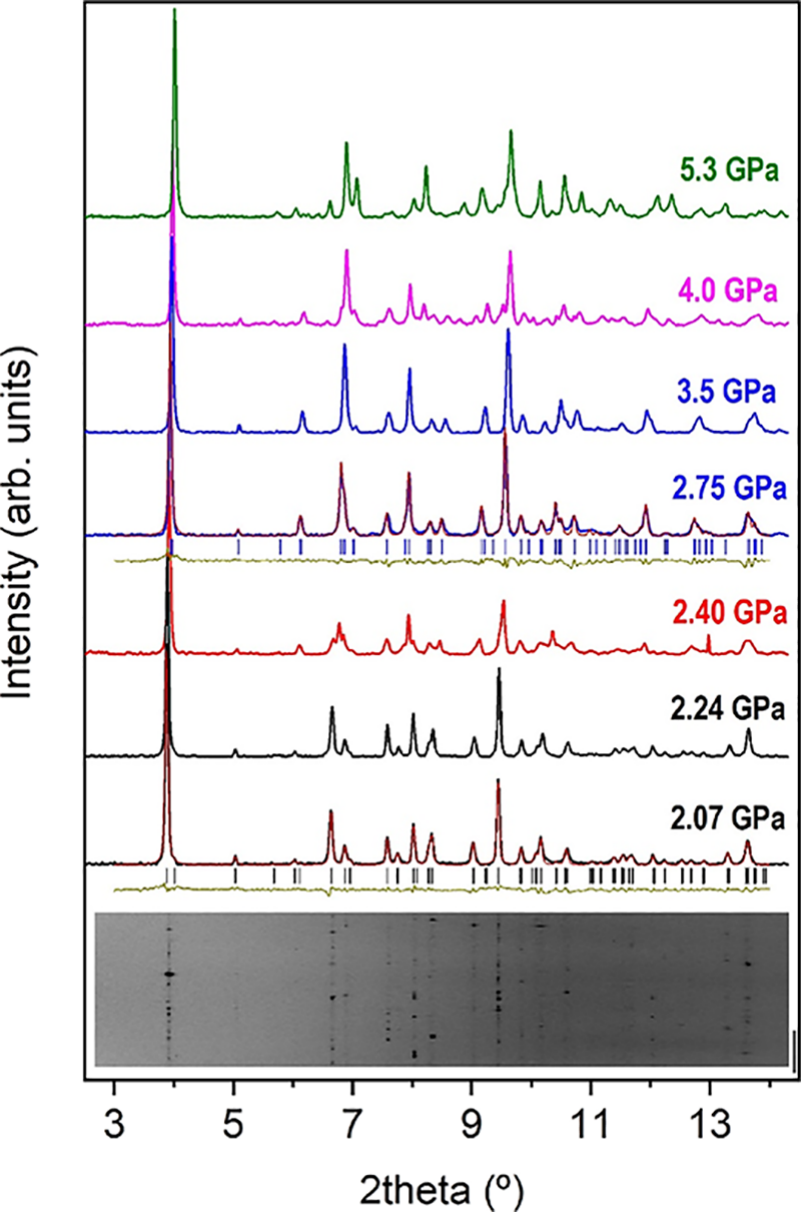
Selected room-temperature high-pressure X-ray diffraction patterns of nesquehonite up to 5.3 GPa measured using Ne as a pressure transmitting medium. Backgrounds have been subtracted. XRD patterns of nesquehonite, HP1, and HP2 phases are represented in black, blue, and green lines. Reflections are indicated as vertical marks. LeBail refinements of nesquehonite at 2.07 GPa and the HP1 phase at 2.75 are shown. At the bottom, the cake image of the raw data of HP nesquehonite is shown. The synchrotron radiation wavelength is 0.4246 Å.

**Figure 3:**
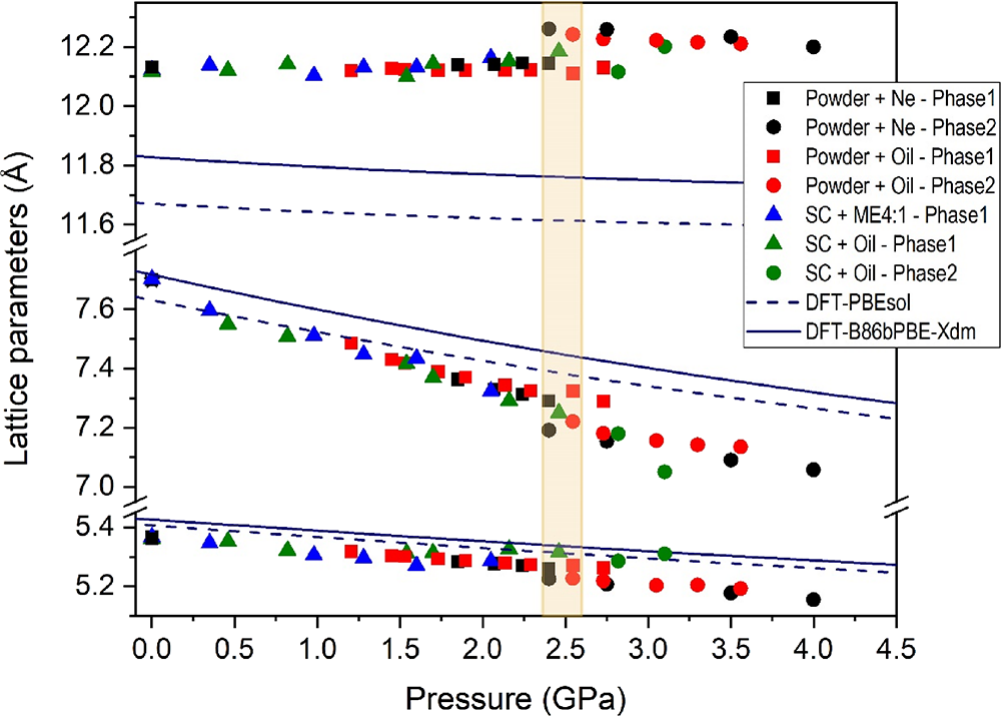
Pressure dependence of the lattice parameters up to 4 GPa. Solid symbols correspond to experimental data. The color codes given in the figure inset indicate the experimental run (powder or single-crystal, pressure transmitting medium; phase 1 = nesquehonite; phase 2 = HP1). Solid and dashed blue lines correspond to DFT calculations using the PBEsol and B86bPBE-XDM functionals, respectively.

**Figure 4:**
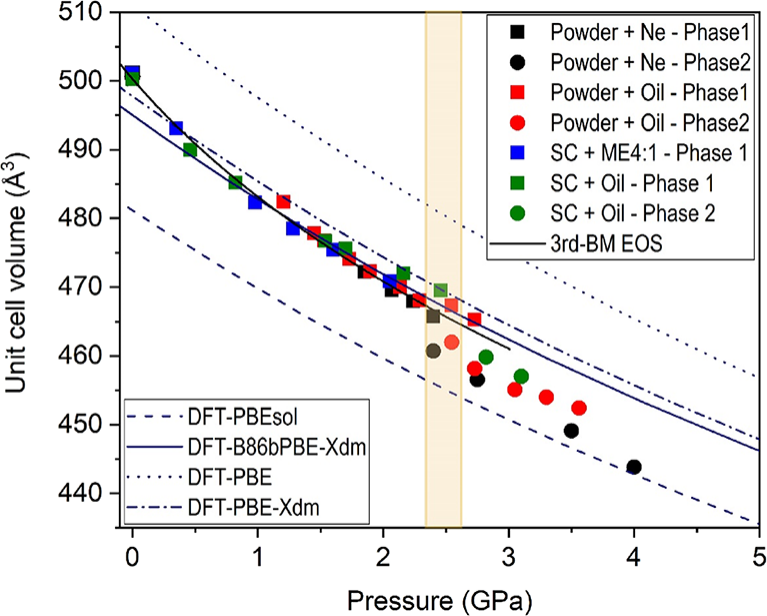
Pressure dependence of the unit cell volume up to 4 GPa. Solid symbols correspond to experimental data. The color codes given in the figure inset indicate the experimental run (powder or single-crystal, pressure transmitting medium; phase 1 = nesquehonite; phase 2 = HP1). Solid and dashed blue lines correspond to DFT calculations using the PBEsol and B86bPBE-XDM functionals, respectively. The solid black line represents the fit of the nesquehonite *P*–*V* data to a third-order Birch–Murnaghan equation of state.

Powder diffraction data present intensities that do not correspond to perfect homogeneous and randomly oriented powder (Figure 2), so only peak positions and not relative intensities could be used in the structural analysis. Therefore, from powder diffraction data, we could accurately infer the lattice parameters of MgCO_3_·3H_2_O nesquehonite upon compression. Powder XRD patterns at different pressures using Ne and silicone oil as pressure transmitting media are shown in Figure 2 and Figures S1–S3 (Supporting Information). Indexations and profile fittings of the powder XRD patterns suggest that the structure can be described by the monoclinic *P*2_1_/*n* space group up to 4.0(3) GPa, when the initial diffraction peaks are replaced by new ones, indicating the existence of a phase transition. However, the evolution of the lattice parameters (Figure 3) and unit cell volumes (Figure 4) as a function of pressure also evidences a clear discontinuity at 2.4(2) GPa. More specifically, according to our experiments, the *a* and *b* axes suddenly decrease by 1.4 and 0.8%, respectively, at this first transition, while the *c* axis increases by about 0.9%, and the monoclinic β angle does not change noticeably, giving as a result a unit cell contraction of about 1.1%. Henceforth, we denote this first high-pressure phase as HP1. It is worth noting that the low symmetry and the large unit cell parameters preclude an unequivocal space group assignment since numerous reflections overlap in powder XRD patterns, and this problem is accentuated upon compression with the progressive broadening of the XRD reflections. Single-crystal XRD measurements allowed us to characterize the progressive transformations of the nesquehonite structure with increasing pressure and tentatively determine (because the crystal diffraction data largely deteriorate upon compression) the nature of the HP1 phase.

**Figure 5:**
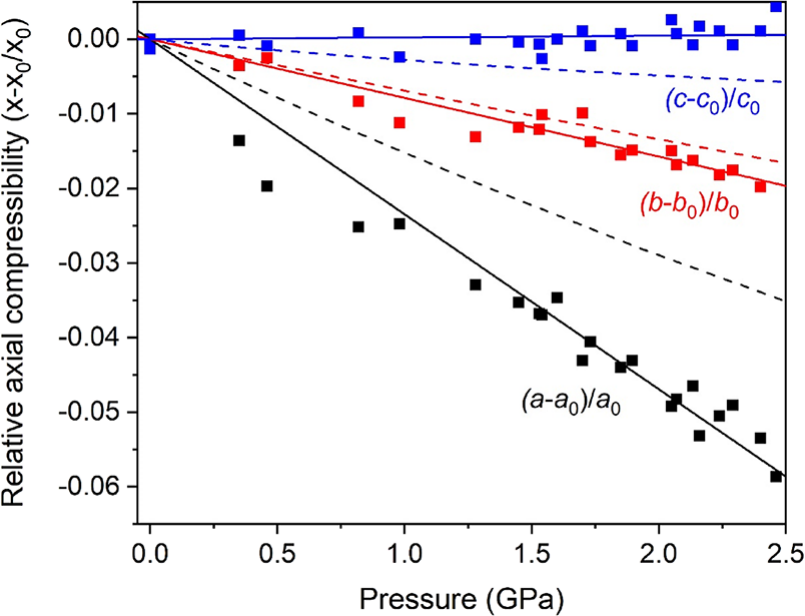
Relative axial compressibility of MgCO_3_·3H_2_O nesquehonite at room temperature, where the anisotropic behavior is clearly shown. Black, red, and blue square symbols correspond to (*a* – *a*_0_)/*a*_0_, (*b* – *b*_0_)/*b*_0_, and (*c* – *c*_0_)/*c*_0_ experimental data, respectively. The solid and dashed blue lines correspond to linear fits to experimental data and results from B86bPBE-XDM calculations, respectively.

**Figure 6:**
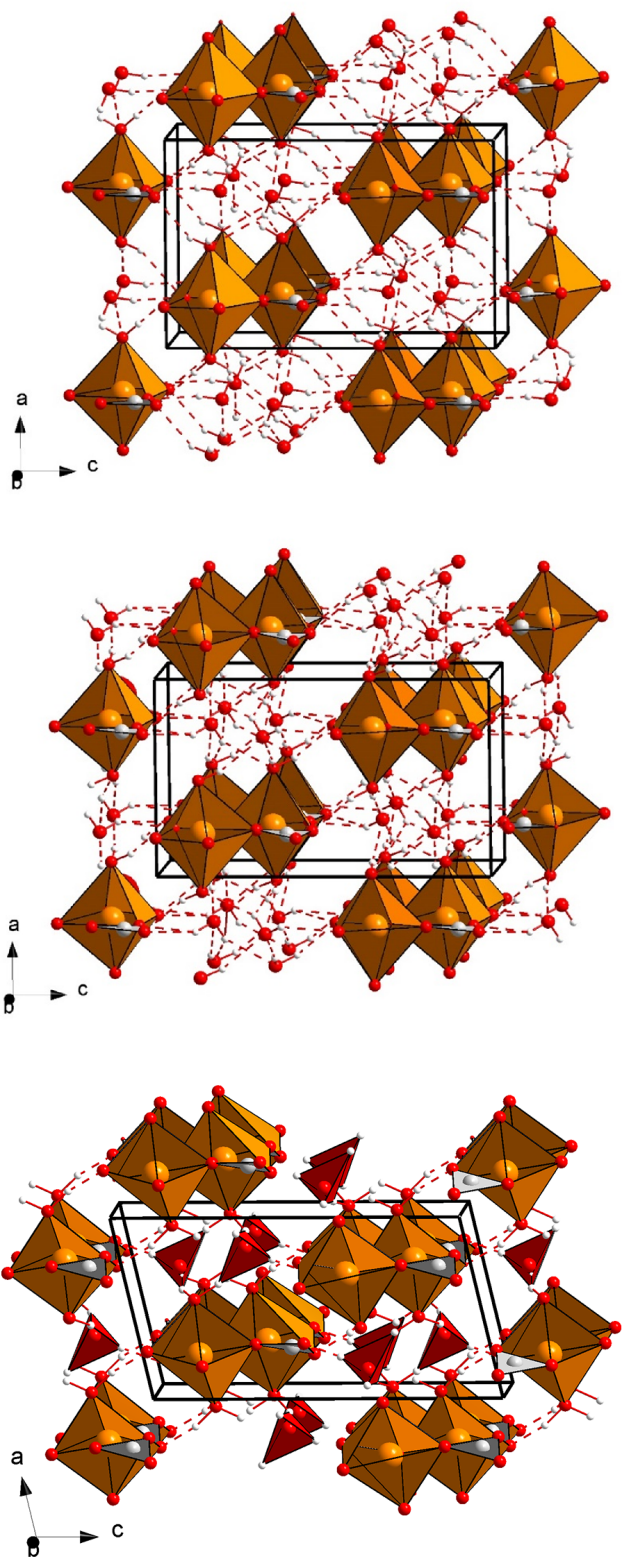
Schematic view of (top) the nesquehonite structure, (center) the HP1 structure, and (bottom) the theoretically predicted HP structure at 18 GPa. Orange, red, gray, and white spheres correspond to the Mg, O, C, and H atoms, respectively. H-bonds are represented as dashed contacts. The [MgO_6_] octahedra and [CO_3_] triangular groups are illustrated. Formed [OH_4_] groups are also shown in the bottom figure.

We studied the compressibility and anisotropy of the initial nesquehonite structure at room temperature. Taking into account the description previously made, intuition suggests that this structure should be stiffer along the *b* axis than along the other two axes, with no obvious indication that either the *a* axis or the *c* axis should behave in any unusual fashion (Figure 1). Experimentally, we found that the lattice parameters of the monoclinic unit cell (*a*, *b*, *c*, and β) vary smoothly with increasing pressure between 0 and 2.4(2) GPa (Figures 3 and 4), which supports the absence of any phase transition in this pressure range. Nesquehonite lattice parameters and unit cell volumes at different pressures are collected in Table S2 of the Supporting Information. Tables S3 and S4 summarize the parameters characterizing the single-crystal data collection and refinement. The axial compressibilities, defined as κ = −1/*x*(∂*x*/∂*P*) (where *x* = *a*, *b*, and *c*), estimated from all our experimental (theoretical) data are κ_*a*0_ = 21.6(7) × 10^–3^ GPa^–1^ (14.2(3) × 10^–3^ GPa^–1^), κ_*b*0_ = 7.5(4) × 10^–3^ GPa^–1^ (6.66(7) × 10^–3^ GPa^–1^), and κ_*c*0_ = −8(4) × 10^–4^ GPa^–1^ (2.34(13) × 10^–3^ GPa^–1^), which evidence the strong anisotropy in this compound. Despite different systematic errors, the results of single-crystal and synchrotron powder XRD measurements using different pressure media are very similar and therefore comparable. The small negative axial compressibility of the *c* axis found experimentally is noteworthy. Materials expanding in one direction during a densification process are rare. Rotation of polyhedra, conformational changes, and an increase in bond angles are among possible mechanisms that account for structure expansion on hydrostatic compression. Sometimes, as in this case, they are associated with the pressure-induced formation of intermolecular H-bonding interactions that change the mechanical properties of the solid.^59−61^ Relative axial compressibilities are represented in Figure 5, where it is also clearly shown that the most compressible axis is the *a* axis and that the *c* axis slightly expands upon compression. Therefore, the assumptions based on intuition at first sight do not work. The axial response to external pressure likely arises from the fact that the H-bonds are highly directed along the *c* axis with H-bond distances in a narrow range at room pressure (between 1.89 and 2.36 Å followed by a gap up to 2.72 Å, Figure 6a). At higher pressures, the distances of the distant O–H contacts progressively decrease until the hydrogen bond distances have an almost continuous distribution. This behavior could be related to the fact that the *c* axis slightly increases upon compression.

A third-order Birch–Murnaghan (BM) EOS was fitted to all our pressure–volume nesquehonite data sets at room temperature, including HP single-crystal and synchrotron powder XRD data (Figure 4), yielding a zero-pressure unit cell volume (*V*_0_), a bulk modulus (*B*_0_), and its first pressure derivative (*B*′_0_) of *V*_0_ = 500.3(7) Å^3^, *B*_0_ = 24(2) GPa, and *B*′_0_ = 12(3), respectively. This experimental result evidences that the bulk modulus rapidly increases upon compression, as in other carbonates with weak atomic interactions.^62,63^ The high compressibility of this compound is confirmed by the values obtained from our calculations: *V*_0_ = 494.8(2) Å^3^, *B*_0_ = 38.2(5) GPa, and *B*′_0_ = 4.43(16).

Despite the serious limitations of XRD measurements to locate H atoms in the structure, the refined single-crystal atomic positions at 2.82 GPa provide a tentative structure of the HP1 phase, as shown in Figure 6b. It is described within the same space group as the initial phase, *P*2_1_/*n*, and the lattice parameters present a small but clear discontinuity with respect to the initial nesquehonite phase. The only significant structural differences come from slight displacements of the O atoms, which make the [CO_3_] carbonate groups not fully perpendicular to the *a* axis, forming as a consequence more irregular [MgO_6_] octahedra (Figure 6b). Our refinement at 2.82 GPa suggests a noticeable increase of the number of H-bonds in the HP1 phase. The compressibility of this phase could not be determined due to the small amount and dispersion of available *P*–*V* data points (Figure 4 and Table S5).

**Figure 7:**
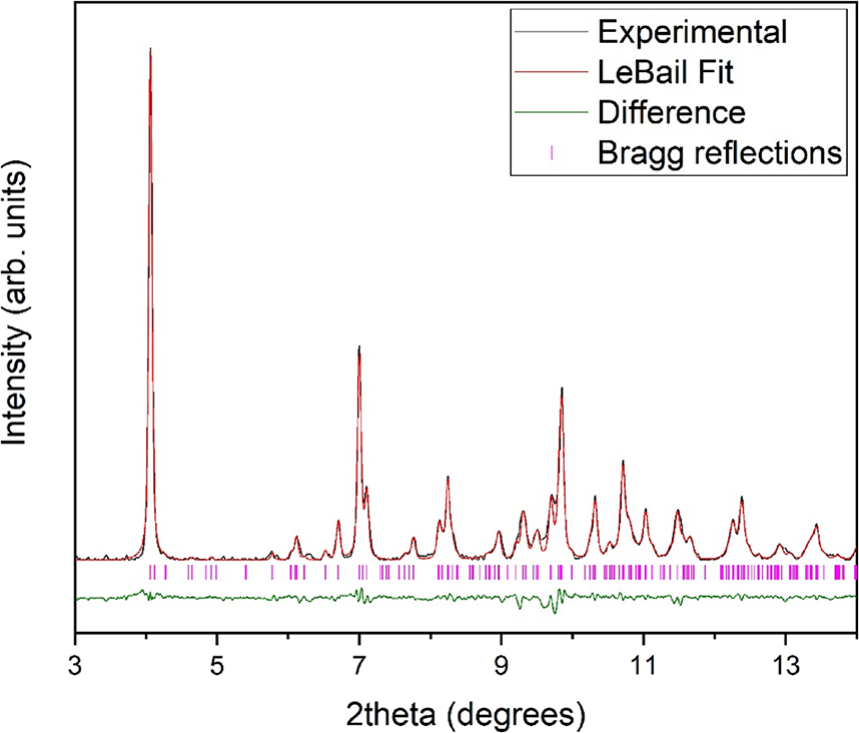
LeBail fit of the integrated XRD pattern of MgCO_3_·3H_2_O at 7.5 GPa and room temperature. Observed, calculated, and difference X-ray diffraction profiles are depicted in black, red, and green, respectively. Magenta vertical marks indicate Bragg reflections. Synchrotron radiation wavelength = 0.4246 Å.

Above 4.0(3) GPa, a significant change in the XRD patterns of dense nesquehonite occurs. However, the interpretation of the new diffraction peaks is difficult due to the limited quality of the XRD patterns and the fact that a pressure-induced phase transformation from an initial trihydrated carbonate was never reported in the literature. Noting the roughly constant relative intensity of the diffraction peaks, we can say that the high-pressure polymorph seems to be stable upon compression from 5.3 up to 18.9 GPa, the maximum pressure reached in this study (see Figure S1 in the Supporting Information). The diffraction peaks at 7.5 GPa could be indexed with a monoclinic unit cell with lattice constants *a* = 9.934(4) Å, *b* = 6.951(3) Å, *c* = 11.864(6) Å, and β = 95.34(5)° (*V* = 815.6.(5) A^3^), which would entail a unit cell content of 8 MgCO_3_·3H_2_O formula units. The LeBail fit to the experimental XRD pattern shown in Figure 7 is excellent. Such a unit cell would imply a volume change of about 2.5% at the transition. We looked for tentative structures using the Endeavor software,^64^ which provides possible structure solutions using a combined global optimization of the difference between calculated and observed diffraction data and of the potential energy of the system,^65^ but without success. New experiments are needed to determine the structure of the HP2 phase of MgCO_3_·3H_2_O.

**Figure 8:**
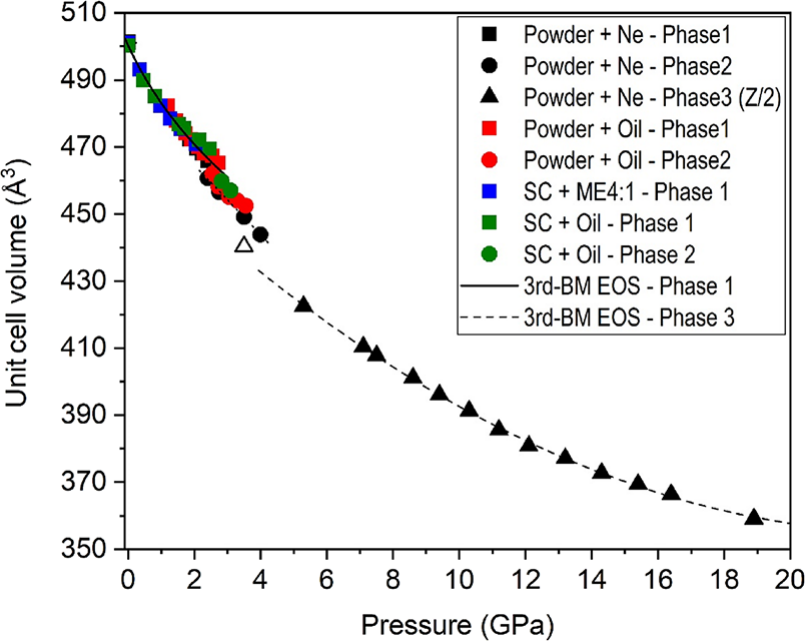
Pressure dependence of the unit cell volume up to 19 GPa. Solid symbols correspond to experimental data. The color codes given in the figure inset indicate the experimental run (powder or single-crystal, pressure transmitting medium; phase 1 = nesquehonite; phase 2 = HP1; phase 3 = HP2). Solid and dashed lines correspond to fits of the nesquehonite and HP2 phase *P*–*V* data to third-order Birch–Murnaghan equations of state.

The equation of state of this second high-pressure HP2 phase was estimated from the indexed unit cell volumes at 12 different pressures (Figure 8). It is defined by the following characteristic parameters: *V*_0_ = 993(25) Å^3^, *B*_0_ = 21(6) GPa, and *B*′_0_ = 5.5(6) (Figure 6). Indexed lattice parameters of this phase at different pressures are also collected in Table S6. The evolution of the indexed lattice parameters of the HP2 phase is represented in Figure S4. These data suggest a change in the compression mechanism at around 12 GPa, which is especially apparent in the *a* lattice parameter, where the slope of the lattice parameter versus pressure appears to slightly change from steeper to more shallow. This is not readily observed in the *b* axis, but the *c* axis and monoclinic angle both show subtle anomalies at this pressure. However, there are no appreciable anomalies in the *P*–*V* curve. This subtle change in compressibility could be related to the difficulties of unequivocally indexing a monoclinic phase with a limited number of single reflection peaks.

In Figure S4, the lattice parameters of the low-pressure phases (up to ∼4 GPa) are also plotted. Nesquehonite and HP1 phases are based on a unit cell with half of the formula units (*Z* = 4). To compare the lattice parameters of the low-pressure phases with the those of the HP2 phase, the HP2 *a* axis is represented with the nesquehonite *b* axis multiplied by 2, so that the low- and high-pressure phases are compared with the same formula units. The HP2 *b* axis and *c* axis are plotted with the *a* axis and *c* axis of the low-pressure nesquehonite phases, respectively. It can be seen that the lattice dimensions are comparable, which suggests that, despite the volume collapse and the lattice discontinuities at the second pressure-induced transition, the topology of the HP2 phase could be related to that of initial nesquehonite.

Interestingly, a dense phase that does not explain the experimental XRD patterns is predicted by DFT calculations above 15 GPa (theoretical *P*–*V* evolution in Figure S5 of the Supporting Information). This theoretical HP phase can also be described by a *P*2_1_/*n* space group, such as nesquehonite and the HP1 phase. The structure of this theoretical dense phase is represented in Figure 6c, which can be compared with experimental phases. Its cif file with the lattice parameters and atomic coordinates can be found in the Supporting Information. According to the calculations, the formation of this predicted polymorph would entail discontinuities in the evolution of the lattice dimensions with a contraction of the *b* axis of ∼3.5%, an expansion of the *c* axis of ∼4.3%, and an increase of almost 9° in the monoclinic β angle, while the *a* axis maintains a similar length. As a consequence of the change in lattice parameters and atomic coordinates, the water molecules surrounded by other water molecules present H–O distances between 1.37 and 1.6 Å, significantly shorter than that of an H-bond. Despite the obvious differences between the experimental HP1 phase and this one, it is striking that the [CO_3_] groups of both structures are inclined with respect to the *bc* plane, producing the same kind of deformation in the Mg-centered octahedral units, which is more pronounced in the theoretical predicted phase.

The initial nesquehonite phase was experimentally recovered when pressure was released, with lattice parameters similar to the precompressed sample (see Table S2).

### Dense Nesquehonite at High Temperatures

The mechanism of thermal decomposition of nesquehonite at room pressure in a still air atmosphere is roughly well-understood below 300 °C, with some discrepancies above this temperature.^26,28,66,67^ The differential (DTA) and thermogravimetric (TGA) thermal analyses of nesquehonite reported in the literature show the following features: (i) no weight loss occurs below 52 °C, (ii) a 39% loss of weight occurs by 350 °C, which corresponds to the loss of three water molecules per formula unit, and (iii) further losses related with the decarbonation process take place in the temperature range of 350–530 °C, with the total weight loss of 70.8%. It has been suggested that this decomposition process starts with the formation of an amorphous^67^ or ill-crystallized^28^ carbonate phase at 115 °C with about 2 H_2_O molecules in the formula unit. Subsequently, upon further heating, nesquehonite in a static air atmosphere transforms into hydromagnesite, Mg_5_(CO_3_)_4_(OH)_2_·4H_2_O.^26,67^

We have performed a HT run on nesquehonite, which was initially compressed to 0.7 GPa in the water loss temperature range. The experiments were conducted in a sealed pressure chamber where the sample has no access to air or moisture at a slower heating rate (1°/min) than that used in aforementioned air atmosphere experiments (5–20°/min).^28,67^ Note that time at sustained high temperatures plays a crucial role in phase transformations^68^ since, at high heating rates, heat is dissipated more easily, and hence, decomposition or dehydratation could start at a comparatively higher temperature.

**Figure 9:**
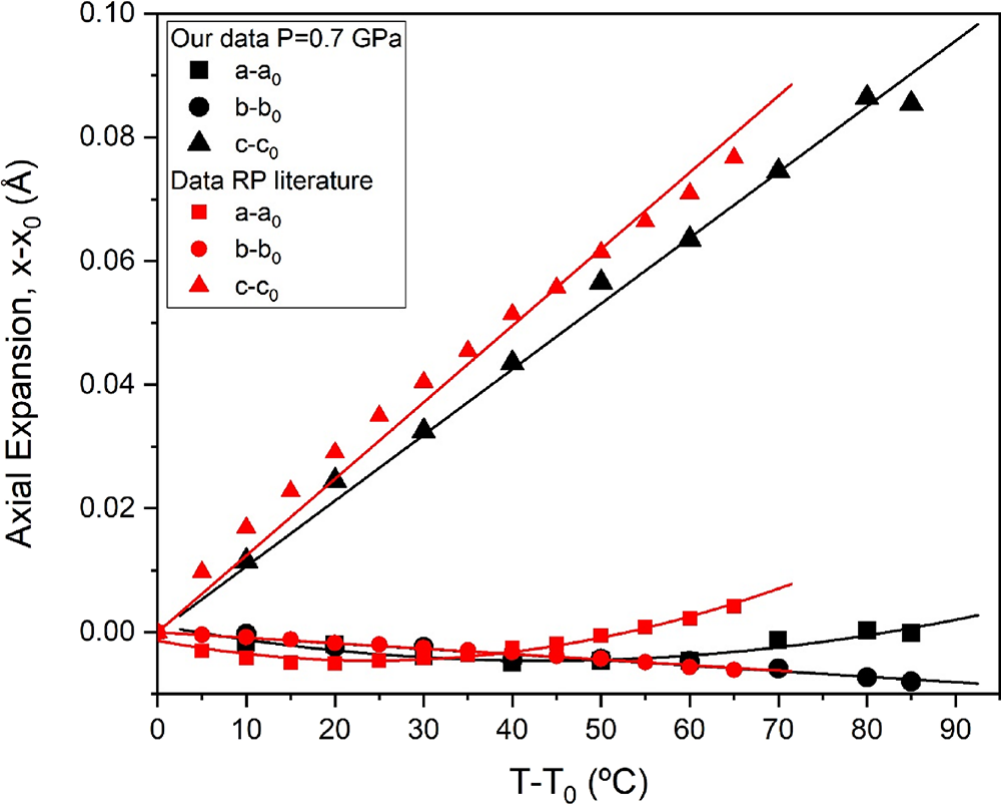
Evolution of the axial change with temperature. Black and red symbols correspond to our experimental data at 0.7 GPa and the results of Ballirano et al. at room pressure.^27^ Squares, circles, and triangles represent (*a* – *a*_0_), (*b* – *b*_0_), and (*c* – *c*_0_), respectively. Linear and polynomial fits are depicted as solid lines.

In this run, we used silicone oil as a quasi-hydrostatic pressure transmitting medium, and the initial nesquehonite phase undergoes a decomposition or structural transformation at 115 °C. This temperature coincides with that reported in the literature for the unknown partially dehydrated poorly crystallized phase at room pressure.^28,67^ The evolution of the unit cell parameters of nesquehonite at 0.7 GPa with increasing temperature and its comparison with reported data at room pressure^27^ are depicted in Figure 9. Despite a smooth increase of the volume, the unit cell axes show a markedly anisotropic behavior, quite similar to that reported at room pressure. Axial thermal expansion is positive only for the *c* axis, which regularly expands upon heating. The experimentally obtained values for the *c* axis thermal expansion coefficient at room pressure and 0.7 GPa are α_*c*_ = 1.24(2) × 10^–3^ and 1.06(2) × 10^–3^ K^–1^, respectively. The *b* axis contracts at high temperature, following a roughly linear behavior with a thermal expansion coefficient of α_*b*_ = −8.8(13) × 10^–5^ and −9.0(3) × 10^–5^ K^–1^ at room pressure and 0.7 GPa, respectively. The *a* cell parameter decreases slightly up to 50 and 70 °C for room and HP runs, respectively, and subsequently increases (see Figure 9).

**Figure 10:**
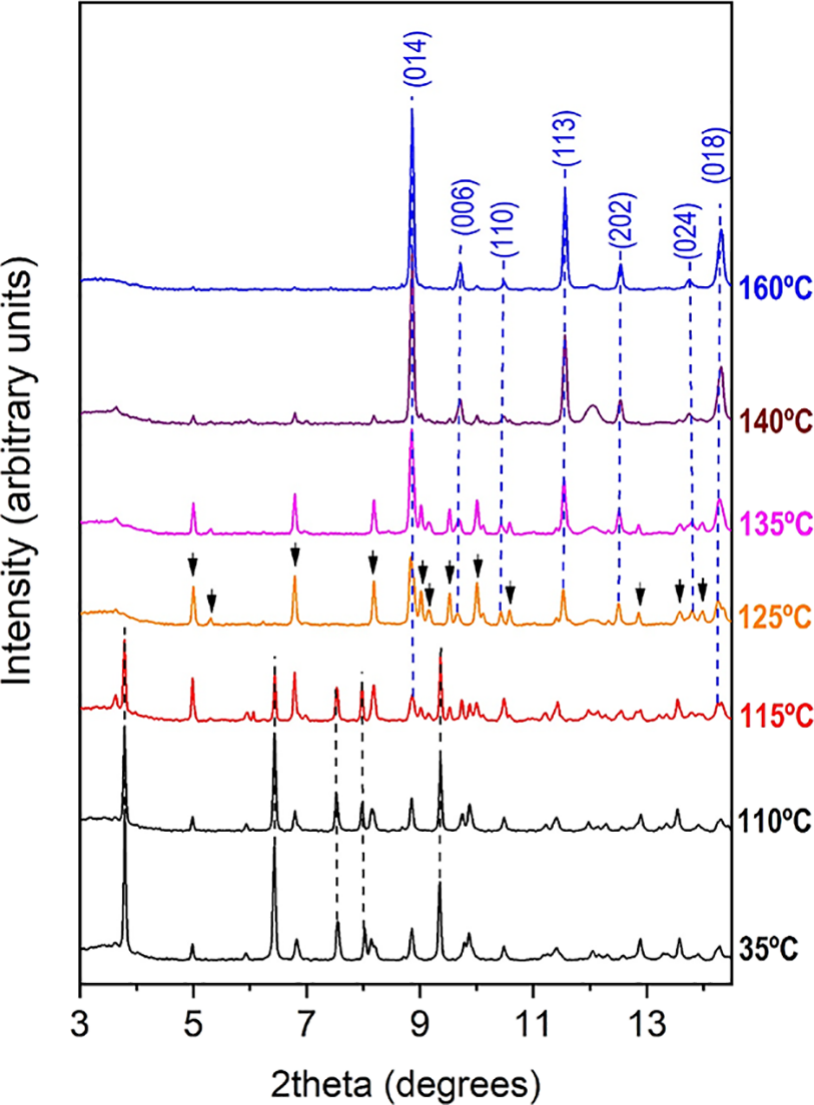
Selected high-temperature X-ray diffraction patterns of nesquehonite up to 160 °C measured using silicone oil as a pressure transmitting medium. Black and blue dashed lines mark the position of the most intense reflections of MgCO_3_·3H_2_O nesquehonite and MgCO_3_ magnesite, respectively. Arrows indicate the position of the reflections associated with the novel HT phase. The synchrotron radiation wavelength is 0.4246 Å.

The XRD patterns above 115 °C reveal the existence of a new phase (Figure 10). Unlike previous room pressure experiments, where only broad bands and low-intensity reflections were observed above that temperature,^27^ we obtained a good-quality XRD pattern with well-defined diffraction peaks. According to the phase diagram of ice/water, at 0.7 GPa, H_2_O is a liquid above room temperature. Therefore, released water does not contribute with diffraction peaks to the XRD pattern at this pressure and high temperatures. A close examination to the XRD patterns between 115 and 140 °C reveals that the intensity of some of the new diffraction peaks progressively decreases, while other reflections, identified as MgCO_3_ magnesite reflections, smoothly increase. At 145 °C, the magnesite reflections get stronger, and at 160 °C, the maximum temperature reached in this run, the XRD pattern mostly corresponds to magnesium carbonate (see Figure 10). In other words, at 0.75 GPa and 160 °C, the novel hydrated HT phase loses all of the water molecules and transforms into MgCO_3_ magnesite.

**Figure 11:**
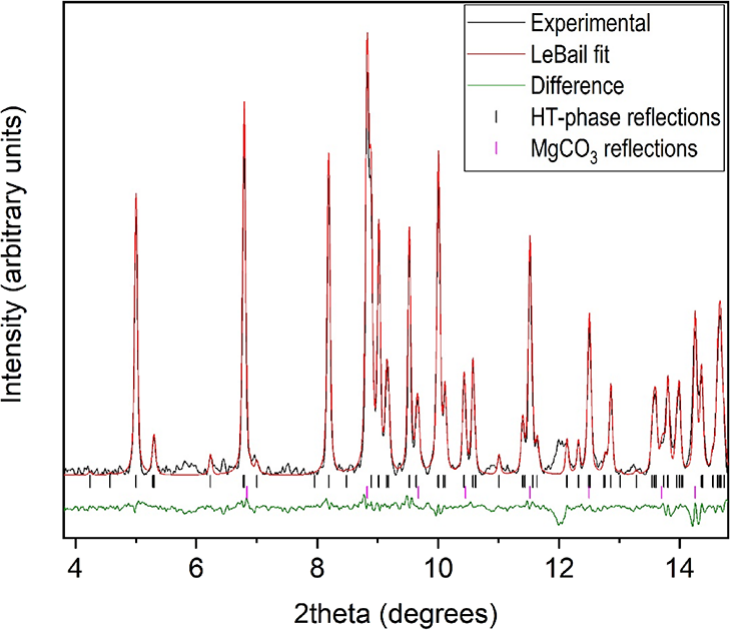
LeBail refinement of the HP-HT XRD pattern of MgCO_3_·3H_2_O at 0.7 GPa and 120 °C. Observed, calculated, and difference X-ray diffraction profiles are depicted in black, red, and green, respectively. Black and magenta vertical marks indicate Bragg reflections of the HT phase and magnesite, respectively. The synchrotron radiation wavelength is 0.4246 Å.

We do not know the exact amount of water molecules present in the novel HT phase. The DTA and TGA analyses at room pressure show that at a temperature of 115 °C, the first endothermic DTA peak occurs together with an approximate loss of weight of 13%, which corresponds to one water molecule per formula unit. On the other hand, our diffraction results show the appearance of magnesite above 115 °C, and the indexation of the rest of the peaks suggests the formation of an orthorhombic structure. The indexation process of 13 reflections at 120 °C provides a unit cell with lattice parameters *a* = 9.183(6) Å, *b* = 5.736(2) Å, and *c* = 5.324(6) Å (*V* = 280.4(4) Å^3^) and a high figure of merit *M*(13) = 42.2. The LeBail refinement of the XRD pattern at this temperature is shown in Figure 11. This solution would imply a volume increase of approximately 14% per formula unit. All of these results suggest that a reaction of the following type could take place at 0.75 GPa and 115 °C,
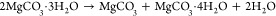
with a subsequent continuous release of water molecules from the novel HT-hydrated phase upon further heating, until complete dehydration and magnesite formation were achieved. Further experiments are needed to confirm this tentative explanation.

## Conclusions

**Figure 12:**
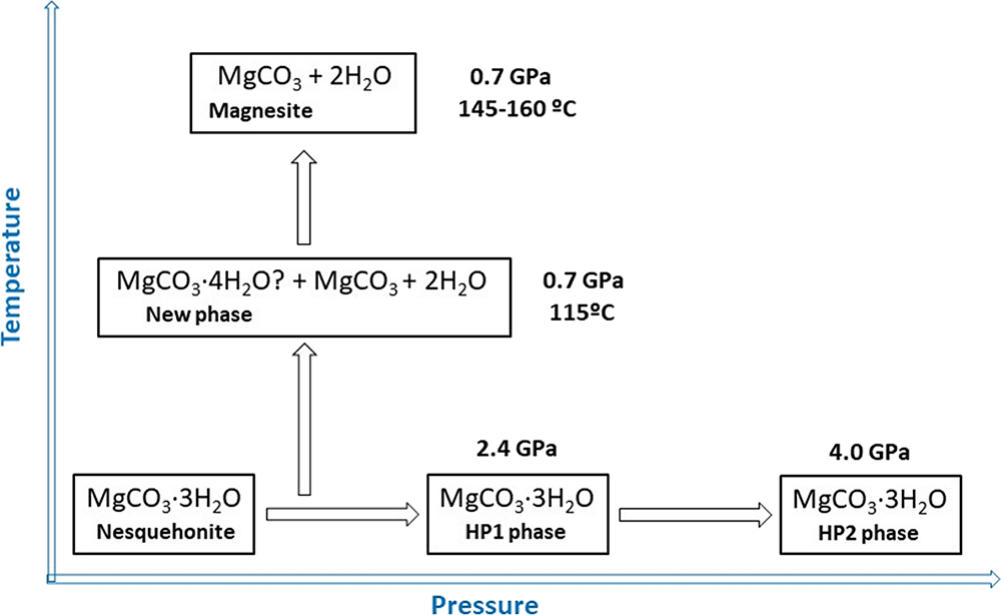
Scheme summarizing the experimental phase transitions and dehydration products observed in this study. Two room-temperature pressure-induced phase transitions and the existence of a novel high-temperature hydrated magnesium carbonate are reported.

Significant progress has been reported in the CO_2_-capture mineralization processes that produce Mg carbonates. The formation of nesquehonite MgCO_3_·3H_2_O is one of the most energy-efficient products of CO_2_ mineralization because its synthesis requires near ambient conditions.^69^ Despite the importance of the phase diagram of this hydrated carbonate to understand (i) the role of water in CO_2_ sequestration methods, (ii) the properties of nesquehonite-based cementitious products, and (iii) the fate of deep carbon upon subduction of carbonate minerals, its stability and structural properties under high pressure (HP) and high temperature (HT) were poorly characterized. In this paper, we have undertaken a joint experimental and computational study of the phase stability and structural behavior of nesquehonite under high pressure and high temperature. For this, we used a combination of single-crystal and synchrotron powder X-ray diffraction (XRD) using resistive-heated diamond anvil cells (DAC) with density functional theory (DFT) calculations. A scheme summarizing the experimental phase transitions and dehydration products observed in the present study can be found in Figure 12.

Our experimental results confirm that the ambient-condition structure is *P*2_1_/*n* monoclinic, and compression experiments at room temperature show that this mineral undergoes two phase transitions at 2.4 (to phase HP1) and 4.0 GPa (phase HP2). Single-crystal X-ray diffraction at 2.8 GPa shows that HP1 has the same space group as the ambient-condition phase, but the structure presents tilted [CO_3_] groups. One important result is that the *c* axis of the ambient-condition phase has a small negative compressibility up to 2.4 GPa, i.e., it expands on increasing pressure, which is probably related to the existence of hydrogen bonds aligned with this axis. Due to peak broadening, peak overlap, and orientation effects, the structure of the HP2 phase could not be determined, but a tentative unit cell was proposed, and its compressibility was estimated. The original nesquehonite structure was recovered on decompression.

Our heating experiments at 0.7 GPa evidenced that nesquehonite has negative thermal expansion coefficients of the *a* axis up to 70 °C and the *b* axis up to the decomposition temperature. They also show the existence of a temperature-induced decomposition of nesquehonite at 115 °C. Although the nature of the high-temperature phase is unknown, the powder diffraction patterns could be indexed with a unit cell whose volume suggests that nesquehonite transforms into a MgCO_3_·4H_2_O phase plus magnesite. All water molecules are lost on further heating, and the sample turns into magnesite at 160 °C. Further structural studies are needed to fully characterize the atomic arrangement in the novel HT-hydrated and HP2 phases.

## Supporting Information Available

Tables with experimental XRD results and additional experimental details and figures showing experimental and theoretical results (PDF)
