# Correction to “Polymorphism and Phase Stability of Hydrated Magnesium Carbonate Nesquehonite MgCO_3_·3H_2_O: Negative Axial Compressibility and Thermal Expansion in a Cementitious Material”

**DOI:** 10.1021/acs.cgd.5c00115

**Published:** 2025-02-12

**Authors:** David Santamaría-Pérez, Raquel Chuliá-Jordán, Javier Gonzalez-Platas, Alberto Otero-dela-Roza, Javier Ruiz-Fuertes, Julio Pellicer-Porres, Robert Oliva, Catalin Popescu

The reference of one funding project was incorrectly indicated.

In the first paragraph of the Acknowledgments where it appears “PGC2021-125518NB-I00” it should be “PID2021-125518NB-I00”.

