# Supplementary material for: Polymorphism and Phase Stability of Hydrated Magnesium Carbonate Nesquehonite MgCO3·3H2O: Negative Axial Compressibility and Thermal Expansion in a Cementitious Material
Source: Cryst Growth Des. 2024 Jan 24;24(3):1159–69. doi: 10.1021/acs.cgd.3c01171 (PMC10854074; doi:10.1021/acs.cgd.3c01171)
Supplement: Supplementary file 1 — cg3c01171_si_001.pdf [file cg3c01171_si_001.pdf]

Supplementary Material

of

Polymorphism and phase stability of hydrated magnesium carbonate nesquehonite $MgCO_3 \cdot 3H_2O$: Negative axial compressibility and thermal expansion in a cementitious material.

D. Santamaría-Pérez[1,*], R. Chuliá-Jordán[2], J. Gonzalez-Platas[3], A. Otero-de-la-Roza[4], J. Ruiz-Fuertes[5], J. Pellicer-Porres[1], R. Oliva[6], C. Popescu[7]

[1] Departamento de Física Aplicada-ICMUV, Universitat de València, MALTA Consolider Team, 46100 Valencia, Spain
[2] Departamento de Didáctica de las Ciencias Experimentales y Sociales, Universitat de Valencia, 46022, Valencia, Spain
[3] Departamento Física. Instituto Universitario de Estudios Avanzados en Física Atómica, Molecular y Fotónica (IUDEA). MALTA Consolider Team. Universidad de La Laguna, 38204, Tenerife, Spain.
[4] Departamento de Química Física y Analítica, Facultad de Química, Universidad de Oviedo, MALTA Consolider Team, 33006, Oviedo, Spain
[5] DCITIMAC, Universidad de Cantabria, MALTA Consolider Team, 39005, Santander, Spain
[6] GEO3BCN – Geosciences Barcelona, CSIC, 08028, Barcelona, Spain
[7] CELLS-ALBA Synchrotron Light Facility, Cerdanyola del Vallés, 08290, Barcelona, Spain

Table S1.- Nesquehonite lattice parameters and atomic coordinates at room conditions from our single-crystal X-ray diffraction measurements and its comparison with DFT calculations and previously reported results (single-crystal SC-XRD [17], neutron powder diffraction NP-XRD [18] in deuterated sample).

	Our exp. SC data			Previous exp. SC-XRD data [17]			Previous exp. NP-XRD data [18]			Our DFT data		
a (Å)	7.7017(8)			7.701(1)			7.72100(12)			7.71705		
b (Å)	5.3606(5)			5.365(1)			5.37518(7)			5.42558		
c (Å)	12.1160(12)			12.126(2)			12.1430(3)			11.82794		
β (º)	90.011(9)			90.41(1)			90.165(4)			91.5870		
V (Å^3)	500.22(9)			501.0(2)			503.956(13)			495.0448		
	x	*y*	*z*	*x*	*y*	*z*	*x*	*y*	*z*	*x*	*y*	*z*
Mg	0.2502(2)	0.0892(3)	0.14987(12)	0.25174(9)	0.08904(6)	0.14970(3)	0.2433(15)	0.0822(9)	0.1466(5)	0.25877	0.08115	0.14706
O1	0.5205(5)	0.0943(7)	0.1505(3)	0.52293(15)	0.0924(2)	0.15188(14)	0.5185(13)	0.0890(17)	0.1570(8)	0.53185	0.08900	0.16276
O2	0.9805(5)	0.0931(8)	0.1492(3)	0.98342(16)	0.0948(2)	0.14742(15)	0.9828(13)	0.0946(17)	0.1490(10)	0.99014	0.07624	0.13993
O3	0.2512(5)	0.4113(6)	0.0620(3)	0.2598(3)	0.41104(13)	0.06205(7)	0.2599(12)	0.4011(10)	0.0644(4)	0.26913	0.40320	0.05411
O4	0.2497(5)	0.2063(6)	0.3069(3)	0.24401(19)	0.20669(13)	0.30691(6)	0.2502(13)	0.1974(10)	0.3096(5)	0.23796	0.19576	0.30921
O5	0.2499(4)	0.8165(5)	0.0204(2)	0.25136(18)	0.81644(13)	0.02004(6)	0.2519(13)	0.8069(10)	0.0202(5)	0.26612	0.81021	0.01274
O6	0.2566(8)	0.3496(9)	0.8378(3)	0.2803(2)	0.3498(2)	0.83840(12)	0.2789(12)	0.3516(14)	0.8305(6)	0.30023	0.34161	0.83097
C	0.2496(6)	0.1367(8)	0.4085(4)	0.2440(3)	0.13800(17)	0.40863(8)	0.2524(10)	0.1381(10)	0.4084(4)	0.23482	0.12929	0.41427
H1	0.557	0.150	0.089	0.582(3)	0.153(3)	0.100(3)	0.5860(11)	0.1569(14)	0.0879(7)	0.59311	0.16675	0.09883
H2	0.558	0.945	0.153	0.575(3)	0.967(5)	0.158(2)	0.5852(11)	0.9536(15)	0.1568(7)	0.58879	0.92236	0.16737
H3	0.911	0.139	0.099	0.920(3)	0.155(4)	0.093(3)	0.9109(14)	0.1472(16)	0.0931(9)	0.90537	0.13438	0.08117
H4	0.924	0.115	0.209	0.925(3)	0.114(3)	0.206(2)	0.9174(12)	0.1197(20)	0.2172(8)	0.93162	0.08679	0.2129
H5	0.162	0.287	0.814	0.177(4)	0.313(4)	0.829(2)	0.1638(11)	0.3108(16)	0.8211(8)	0.18462	0.32879	0.79369
H6	0.240	0.368	0.907	0.273(4)	0.378(3)	0.904(2)	0.2733(13)	0.3739(13)	0.9184(6)	0.28262	0.37805	0.91309

Table S2.- Lattice parameters and unit-cell volumes of nesquehonite up to 2.7 GPa at room temperature.

Pressure (GPa)	*a* axis (Å)	*b* axis (Å)	*c* axis (Å)	β angle (º)	Volume (Å^3)
10^{-4}(pwd)	7.696(1)	5.361(2)	12.125(2)	90.28(2)	500.3(1)
10^{-4} (pwd-recovered)	7.699(2)	5.366(3)	12.132(4)	90.31(4)	501.2(2)
10^{-4} (SC-Met)	7.7019(6)	5.3663(4)	12.1239(9)	90.048(7)	501.09(7)
10^{-4} (SC-Oil)	7.7017(8)	5.3606(5)	12.1160(12)	90.011(9)	500.22(9)
0.35 (SC-Met)	7.597(6)	5.3471(5)	12.138(4)	90.04(6)	493.1(4)
0.46 (SC-Oil)	7.550(10)	5.3530(9)	12.121(7)	90.21(10)	489.9(7)
0.82 (SC-Oil)	7.508(10)	5.3216(9)	12.142(6)	90.09(10)	485.2(7)
0.98 (SC-Met)	7.511(15)	5.3060(12)	12.103(10)	89.81(15)	482.3(10)
1.21 (pwd-Oil)	7.485(2)	5.317(3)	12.120(4)	90.46(4)	482.3(2)
1.28 (SC-Met)	7.448(17)	5.2958(14)	12.132(11)	90.18(16)	478.5(12)
1.45 (pwd-Oil)	7.430(2)	5.303(3)	12.127(4)	90.50(4)	477.8(2)
1.53 (pwd-Oil)	7.419(2)	5.301(3)	12.123(4)	90.46(4)	476.8(2)
1.54 (SC-Oil)	7.417(11)	5.3121(10)	12.100(8)	90.78(11)	476.7(8)
1.6 (SC-Met)	7.435(18)	5.2703(13)	12.132(12)	89.52(18)	475.4(13)
1.7 (SC-Oil)	7.370(2)	5.3134(19)	12.145(16)	90.3(2)	475.6(16)
1.73 (pwd-Oil)	7.389(2)	5.293(3)	12.121(4)	90.32(4)	474.1(2)
1.85 (pwd-Ne)	7.363(1)	5.283(2)	12.140(2)	90.21(3)	472.2(1)
1.90 (pwd-Oil)	7.370(2)	5.286(3)	12.121(4)	90.37(4)	472.2(2)
2.05 (SC-Met)	7.323(18)	5.286(2)	12.163(14)	90.29(19)	470.8(13)
2.07 (pwd-Ne)	7.330(1)	5.276(2)	12.141(2)	90.01(3)	469.5(1)
2.13 (pwd-Oil)	7.343(2)	5.279(3)	12.123(4)	90.47(4)	469.9(2)
2.16 (SC-Oil)	7.292(19)	5.3257(16)	12.153(13)	90.43(19)	472.0(13)
2.24 (pwd-Ne)	7.313(1)	5.269(2)	12.145(2)	90.25(3)	467.9(1)
2.29 (pwd-Oil)	7.324(2)	5.272(3)	12.123(4)	90.52(4)	468.1(2)
2.4 (pwd-Ne)	7.290(1)	5.260(2)	12.145(2)	90.25(3)	465.7(1)
2.46 (SC-Oil)	7.25(3)	5.3158(19)	12.185(14)	90.6(2)	469.5(17)
2.55 (pwd-Oil)	7.323(2)	5.270(3)	12.110(4)	90.70(4)	467.3(2)
2.73 (pwd-Oil)	7.290(2)	5.262(3)	12.130(4)	90.71(4)	465.2(2)

Table S3.- Details of the data collections, refinement results, and structural data obtained from Nesquehonite single-crystal XRD at different pressures using a mixture methanol-ethanol 4:1 as pressure transmitting medium.

	**0.00 GPa**	**0.35 GPa**	**0.98 GPa**	**1.28 GPa**
CCDC				
Formula	CH_6MgO_6	CH_6MgO_6	CH_6MgO_6	CH_6MgO_6
$D_{calc.}$/ g cm^{-3}	1.837	1.864	1.905	1.921
μ/mm^{-1}	0.300	0.304	0.311	0.314
Formula Weight	138.37	138.37	138.37	138.37
Colour	Colourless	Colourless	Colourless	Colourless
Shape	irregular	irregular	irregular	Irregular
Size/mm^3	0.13×0.06×0.05	0.13×0.06×0.05	0.13×0.06×0.05	0.13×0.06×0.05
T/K	293(2)	293(2)	293(2)	293(2)
Crystal System	Monoclinic	Monoclinic	Monoclinic	Monoclinic
Space Group	$P2_1$/n	$P2_1$/n	$P2_1$/n	$P2_1$/n
a/Å	7.7019(6)	7.597(6)	7.511(15)	7.448(17)
b/Å	5.3663(4)	5.3471(5)	5.3030(12)	5.2958(14)
c/Å	12.1239(9)	12.138(4)	12.103(10)	12.132(11)
α/°	90	90	90	90
β/°	90.048(7)	90.04(6)	89.81(15)	90 .18(16)
γ/°	90	90	90	90
V/Å^3	501.09(7)	493.1(4)	482.3(10)	478.5(12)
Z	4	4	4	4
Wavelength/Å	0.71073	0.71073	0.71073	0.71073
Radiation type	Mo K_α	Mo K_α	Mo K_α	Mo K_α
$2\Theta_{min}$/°	6.264	6.326	6.734	6.718
$2\Theta_{max}$/°	52.74	52.51	52.632	52.65
Measured Refl.	1930	1413	964	946
Indep. Refl.	986	346	341	337
R_{int}	0.0153	0.0375	0.0366	0.0264
Parameters	82	45	45	45
Restraints	0	0	0	0
Largest Peak	0.36	0.37	0.28	0.33
Deepest Hole	-0.39	-0.22	-0.25	-0.24
GooF	1.294	1.157	1.081	1.114
wR_2 (all data)	0.1369	0.1655	0.1299	0.1589
wR_2	0.1351	0.1494	0.1187	0.1425
R_1 (all data)	0.0685	0.0822	0.0780	0.0820
R_1	0.0644	0.0585	0.0590	0.0589

	**1.60 GPa**	**2.05 GPa**
CCDC		
Formula	CH_6MgO_6	CH_6MgO_6
$D_{calc.}$/ g cm^{-3}	1.837	1.952
μ/mm^{-1}	0.316	0.319
Formula Weight	138.37	138.37
Colour	Colourless	Colourless
Shape	irregular	Irregular
Size/mm^3	0.13×0.06×0.05	0.13×0.06×0.05
T/K	293(2)	293(2)
Crystal System	monoclinic	Monoclinic
Space Group	$P2_1$/n	$P2_1$/n
a/Å	7.435(18)	7.323(18)
b/Å	5.2703(13)	5.286(2)
c/Å	12.132(12)	12.163(14)
α/°	90	90
β/°	89.52(18)	90 .29(19)
γ/°	90	90
V/Å^3	475.4(13)	470.8(13)
Z	4	4
Wavelength/Å	0.71073	0.71073
Radiation type	Mo K_α	Mo K_α
$2\Theta_{min}$/°	6.718	6.70
$2\Theta_{max}$/°	52.698	41.558
Measured Refl.	1565	250
Indep. Refl.	332	144
R_{int}	0.0506	0.0466
Parameters	45	40
Restraints	1	1
Largest Peak	0.31	0.24
Deepest Hole	-0.25	-0.21
GooF	1.107	1.214
wR_2 (all data)	0.1604	0.2192
wR_2	0.1439	0.1836
R_1 (all data)	0.0905	0.1113
R_1	0.0656	0.0713

Table S4.- Details of the data collections, refinement results, and structural data obtained from Nesquehonite single-crystal XRD at different pressures using a silicone oil as pressure transmitting medium.

	**0.00 GPa**	**0.46 GPa**	**0.82 GPa**	**1.54 GPa**
CCDC				
Formula	CH_6MgO_6	CH_6MgO_6	CH_6MgO_6	CH_6MgO_6
$D_{calc.}$/ g cm^{-3}	1.837	1.876	1.894	1.928
μ/mm^{-1}	0.300	0.306	0.309	0.315
Formula Weight	138.37	138.37	138.37	138.37
Colour	Colourless	Colourless	Colourless	Colourless
Shape	irregular	irregular	irregular	Irregular
Size/mm^3	0.13×0.05×0.03	0.13×0.05×0.03	0.13×0.05×0.03	0.13×0.05×0.03
T/K	293(2)	293(2)	293(2)	293(2)
Crystal System	monoclinic	monoclinic	monoclinic	monoclinic
Space Group	$P2_1$/n	$P2_1$/n	$P2_1$/n	$P2_1$/n
a/Å	7.7017(8)	7.550(10)	7.508(10)	7.417(11)
b/Å	5.3606(5)	5.3530(9)	5.3216(9)	5.3121 (10)
c/Å	12.1160(12)	12.121(7)	12.142(6)	12.100(8)
α/°	90	90	90	90
β/°	90.011(9)	90.21(10)	90.09	90 .78(11)
γ/°	90	90	90	90
V/Å^3	500.22(9)	489.9(7)	485.2(7)	476.7 (8)
Z	4	4	4	4
Wavelength/Å	0.71073	0.71073	0.71073	0.71073
Radiation type	Mo K_α	Mo K_α	Mo K_α	Mo K_α
$2\Theta_{min}$/°	6.268	6.348	6.376	6.404
$2\Theta_{max}$/°	52.742	52.584	52.69	52.73
Measured Refl.	1087	689	699	880
Indep. Refl.	861	344	340	343
R_{int}	0.0107	0.0253	0.0225	0.0323
Parameters	80	40	40	40
Restraints	0	0	0	0
Largest Peak	0.34	0.33	0.44	0.35
Deepest Hole	-0.35	-0.35	0.31	-0.29
GooF	1.343	1.075	1.092	1.187
wR_2 (all data)	0.1428	0.1955	0.1971	0.1793
wR_2	0.1406	0.1592	0.1752	0.1616
R_1 (all data)	0.0688	0.1110	0.1006	0.1106
R_1	0.0640	0.0746	0.0714	0.0792

	**1.70 GPa**	**2.16 GPa**	**2.46 GPa**	**2.82 GPa**
CCDC				
Formula	CH_6MgO_6	CH_6MgO_6	CH_6MgO_6	CH_6MgO_6
$D_{calc.}$/ g cm^{-3}	1.932	1.947	1.958	1.999
μ/mm^{-1}	0.316	0.318	0.320	0.326
Formula Weight	138.37	138.37	138.37	138.37
Colour	Colourless	Colourless	Colourless	Colourless
Shape	irregular	irregular	irregular	Irregular
Size/mm^3	0.13×0.05×0.03	0.13×0.05×0.03	0.13×0.05×0.03	0.13×0.05×0.03
T/K	293(2)	293(2)	293(2)	293(2)
Crystal System	monoclinic	monoclinic	monoclinic	monoclinic
Space Group	$P2_1$/n	$P2_1$/n	$P2_1$/n	$P2_1$/n
a/Å	7.37(2)	7.292(19)	7.25(3)	7.18(3)
b/Å	5.3134(19)	5.3257(16)	5.3158(19)	5.285(2)
c/Å	12.145(16)	12.153(13)	12.185(14)	12.116(15)
α/°	90	90	90	90
β/°	90.3(2)	90.43(19)	90.6(2)	90 .1(2)
γ/°	90	90	90	90
V/Å^3	475.6(16)	472.0(13)	469.5(17)	459.8(19)
Z	4	4	4	4
Wavelength/Å	0.71073	0.71073	0.71073	0.71073
Radiation type	Mo K_α	Mo K_α	Mo K_α	Mo K_α
$2\Theta_{min}$/°	6.71	6.494	6.688	3.363
$2\Theta_{max}$/°	52.49	52.464	46.24	20.813
Measured Refl.	748	733	738	645
Indep.t Refl.	315	322	251	192
R_{int}	0.0373	0.0466	0.0943	0.1466
Parameters	41	41	39	38
Restraints	0	0	0	0
Largest Peak	0.30	0.38	0.72	0.65
Deepest Hole	-0.32	-0.32	-0.43	-0.54
GooF	1.112	1.153	1.339	1.717
wR_2 (all data)	0.2166	0.2596	0.3880	0.4932
wR_2	0.1812	0.2231	0.3232	0.4185
R_1 (all data)	0.1264	0.1504	0.1849	0.2568
R_1	0.0788	0.0948	0.1382	0.1757

Table S3.- Lattice parameters and unit-cell volumes of the first pressure-induced HP1 phase between 2.4 and 4 GPa at room temperature.

**Pressure (GPa)**	***a* axis (Å)**	***b* axis (Å)**	***c* axis (Å)**	**β angle (º)**	**Volume (Å^3)**
2.4 (pdw-Ne)	7.191(2)	5.225(3)	12.261(4)	90.10(4)	460.7(2)
2.55 (pdw-Oil)	7.220(4)	5.227(4)	12.242(6)	89.56(5)	462.0(5)
2.73 (pdw-Oil)	7.181(4)	5.218(4)	12.227(6)	90.39(5)	458.1(5)
2.75 (pdw-Ne)	7.154(2)	5.206(3)	12.258(4)	89.98(4)	456.5(2)
2.82 (SC-Oil)	7.18(3)	5.285(2)	12.116(15)	90.1(2)	459.8(19)
3.05 (pdw-Oil)	7.156(4)	5.203(5)	12.223(7)	89.43(5)	455.1(6)
3.1 (SC-Oil)	7.05(3)	5.310(3)	12.201(16)	89.8(3)	457(2)
3.3 (pdw-Oil)	7.142(4)	5.204(5)	12.215(7)	90.48(6)	454.0(6)
3.5 (pdw-Ne)	7.090(3)	5.178(3)	12.234(5)	89.91(5)	449.1(3)
3.56 (pdw-Oil)	7.136(4)	5.193(5)	12.210(7)	90.41(6)	452.4(6)
4.0 (pdw-Ne)	7.058(3)	5.154(3)	12.200(5)	89.78(5)	443.8(3)

Table S6.- Lattice parameters and unit-cell volumes of the second pressure-induced HP2 phase between 3.5 and 18.9 GPa at room temperature.

**Pressure (GPa)**	***a* axis (Å)**	***b* axis (Å)**	***c* axis (Å)**	**β angle (º)**	**Volume (Å^3)**
3.5 (pwd-Ne-downstroke)	10.299(5)	7.185(4)	11.952(6)	95.24(6)	880.7(6)
5.3 (pwd-Ne)	10.125(5)	7.053(3)	11.885(6)	95.31(5)	845.0(5)
7.1 (pwd-Ne)	9.975(4)	6.968(3)	11.858(6)	95.27(5)	820.7(5)
7.5 (pwd-Ne)	9.934(4)	6.951(3)	11.864(6)	95.34(5)	815.6(5)
8.6 (pwd-Ne)	9.839(4)	6.906(3)	11.859(6)	95.40(5)	802.3(5)
9.4 (pwd-Ne)	9.765(4)	6.871(3)	11.861(7)	95.48(5)	792.2(5)
10.3 (pwd-Ne)	9.690(4)	6.838(3)	11.866(7)	95.58(5)	782.5(5)
11.2 (pwd-Ne)	9.601(4)	6.807(3)	11.858(7)	95.65(5)	771.2(5)
12.1 (pwd-Ne)	9.532(4)	6.778(3)	11.846(7)	95.69(5)	761.6(5)
13.2 (pwd-Ne)	9.473(4)	6.751(3)	11.851(7)	95.71(5)	754.2(5)
14.3 (pwd-Ne)	9.415(4)	6.720(3)	11.844(7)	95.77(5)	745.5(5)
15.4 (pwd-Ne)	9.374(4)	6.694(3)	11.835(7)	95.84(5)	738.8(5)
16.4 (pwd-Ne)	9.335(4)	6.672(3)	11.827(7)	95.88(5)	732.8(5)
18.9 (pwd-Ne)	9.249(4)	6.618(3)	11.797(7)	96.03(5)	718.2(5)

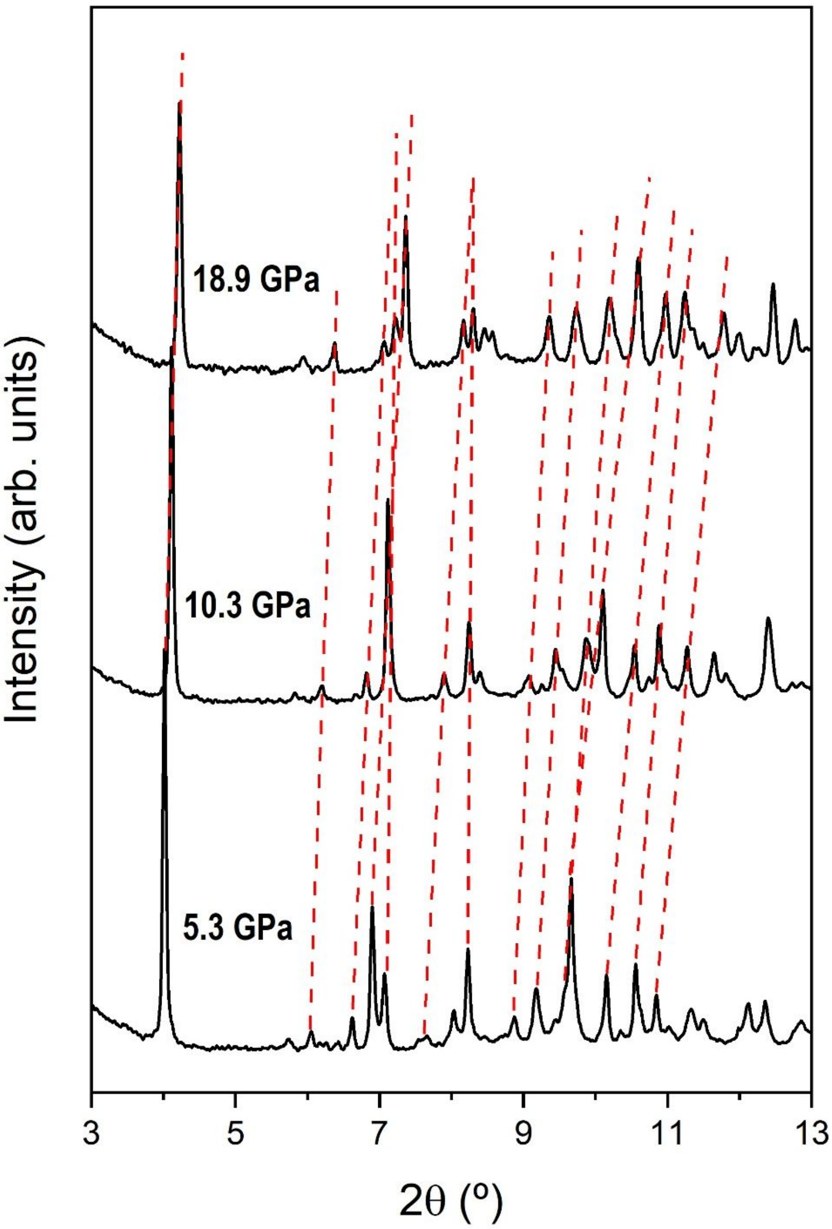

Figure 1S.- Selected XRD patterns of the second pressure-induced HP2 phase upon compression at ambient temperature, using Ne as pressure transmitting medium. The HP2 phase is stable in the 5.3 – 18.9 GPa pressure range. The dashed lines are just a guide to illustrate the angle shift of different reflections with pressure.

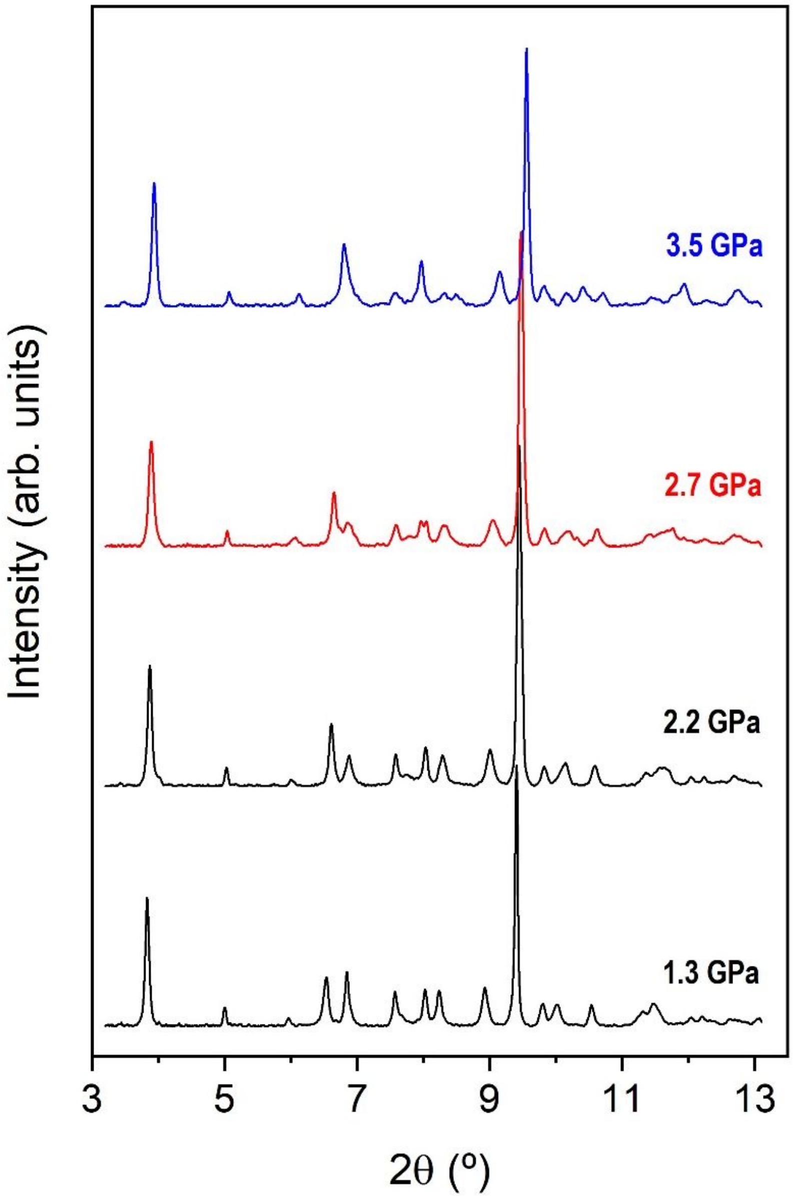

Figure 2S.- Selected XRD patterns of nesquehonite upon compression using silicone oil as pressure transmitting medium, where changes due to the first pressure-induced transition can be observed.

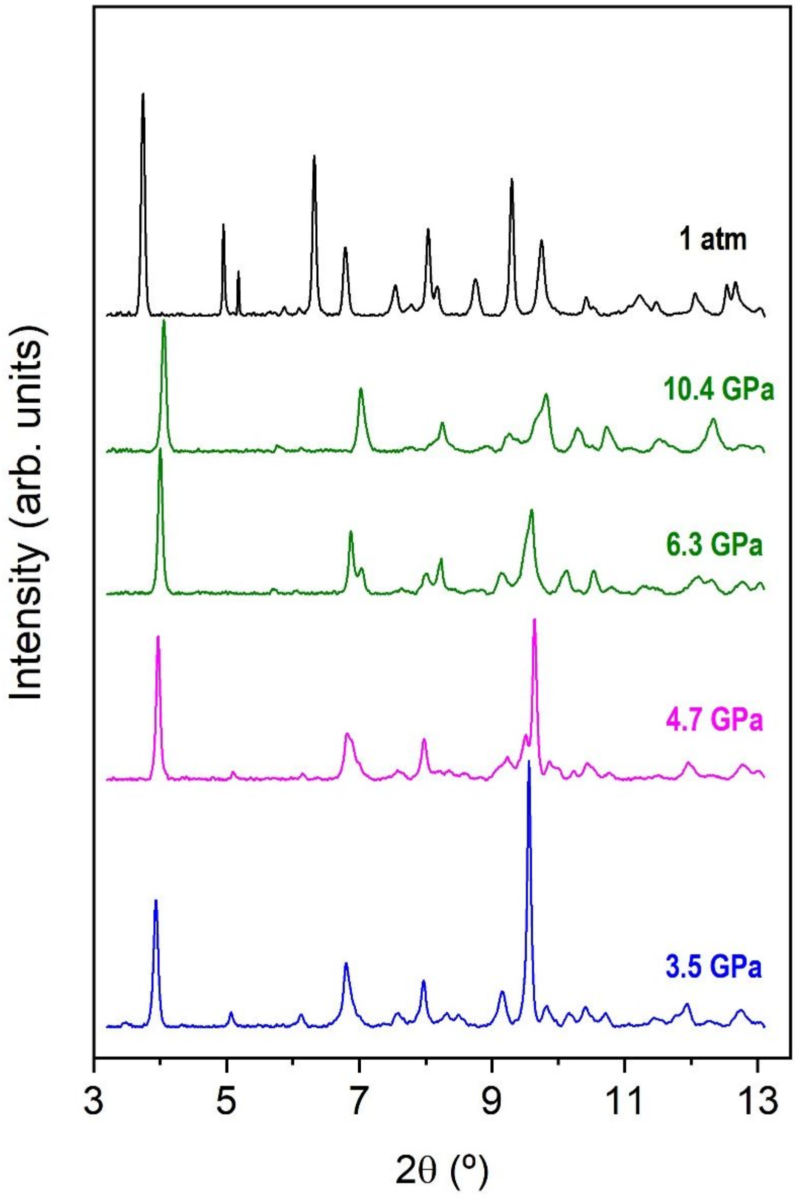

Figure 3S.- Selected XRD patterns of nesquehonite upon compression using silicone oil as pressure transmitting medium, where changes due to the second pressure-induced transition can be observed. The initial nesquehonite structure is recovered after decompression.

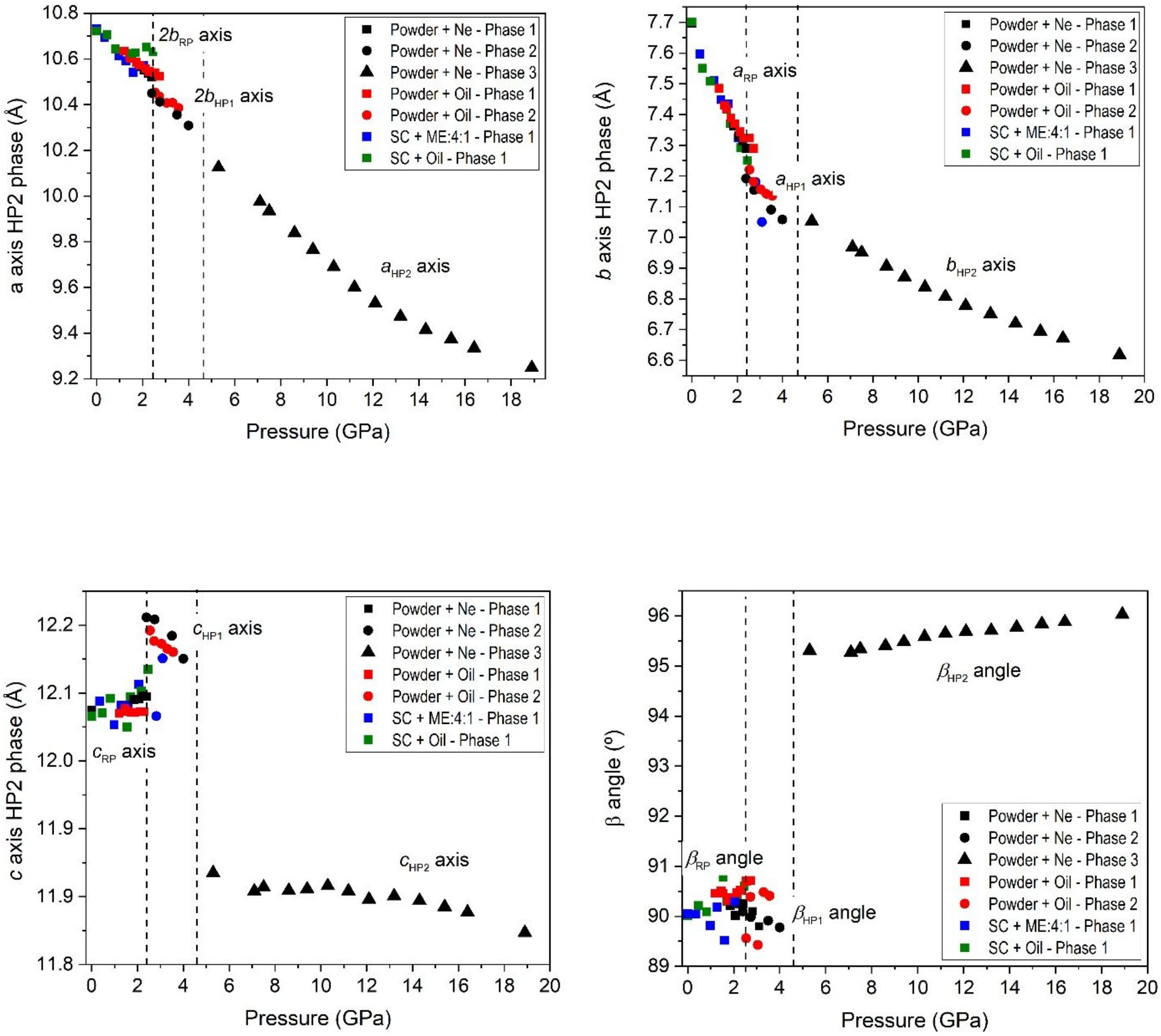

Figure 4S.- Pressure dependence of the lattice parameters of $MgCO_3{\cdot}3H_2O$ up to 19 GPa. The color codes given in the figure inset indicate the experimental run (powder or single-crystal, and pressure transmitting medium; Phase 1 = nesquehonite; Phase 2 = HP1, Phase 3 = HP2). To compare the lattice parameters of the low pressure and HP1 phases with those of the HP2 phase, the low pressure b axis has been multiplied by 2 and plotted with the HP2 *a* axis (so that the different phases are compared with the same formula units. The low pressure *a* axis is represented together with HP2 *b* axis. The *c*-axes and the monoclinic β angles are plotted together.

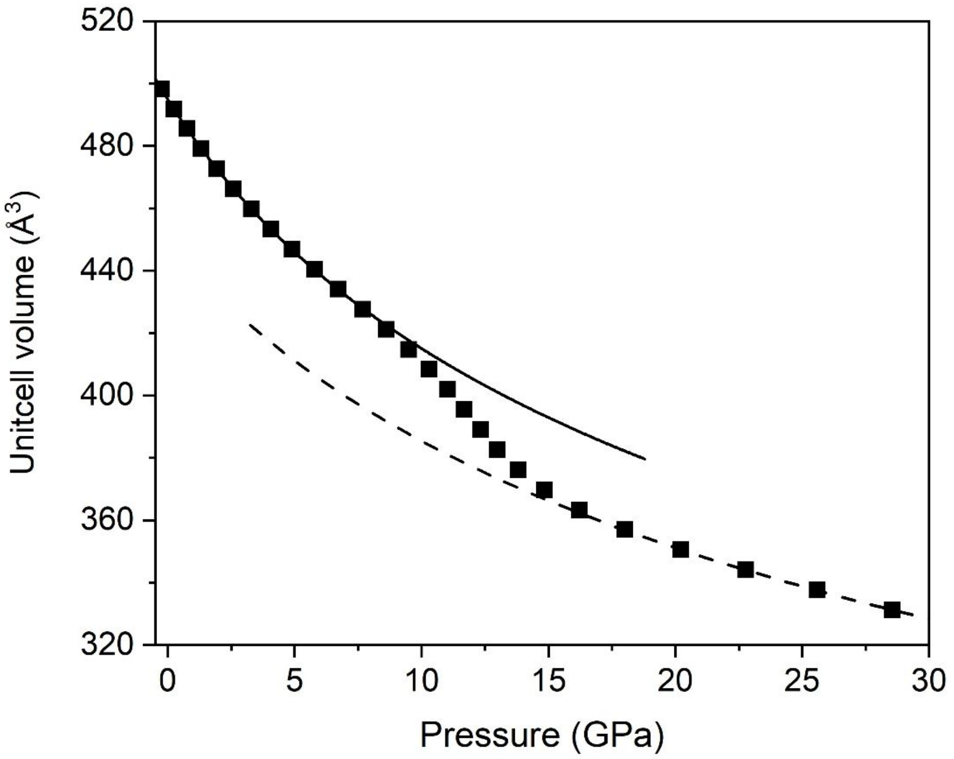

Figure 5S.- Pressure dependence of the unitcell volume according to DFT calculations. A 7% volume collapse is predicted between 10 and 15 GPa due to an atomic rearrangement also described with a $P2_1/n$ space group. Solid and dashed lines show the 3rd-order Birch-Murnaghan EoS fits of P-V DFT-calculated data of nesquehonite and the predicted HP phase.
